# Continuous Infusion Versus Intermittent Bolus Proton Pump Inhibitor Therapy Following Endoscopic Hemostasis for Bleeding Peptic Ulcers: A Systematic Review and Meta-Analysis

**DOI:** 10.3390/jcm15197743

**Published:** 2026-10-08

**Authors:** José Luis Muñoz-Carrillo, Gustavo Bastién-Martínez, Edrei Lopez-Hernandez, Vladimir Guerrero-Toralva, Frida Sofia Ontiveros-Ramos, Alejandra Vargas-Flores, Adair Zabdiel Cortés-Gutiérrez, Francisca Chávez-Ruvalcaba, Natalie Rodríguez-Cortés, Oscar Gutiérrez-Coronado

**Affiliations:** 1 Laboratorio de Inmunología, Centro Universitario de los Lagos, Universidad de Guadalajara, Enrique Díaz de León 1144, Paseos de La Montaña, Lagos de Moreno 47463, Jalisco, Mexico; 2 Centro de Investigación e Innovación Global University (CIIGU), Av Independencia 2134, Trojes de Alonso, Aguascalientes 20116, Aguascalientes, Mexico; 3 Especialidad en Medicina Interna, Universidad Autónoma de Aguascalientes, Av Universidad 940, Ciudad Universitaria, Aguascalientes 20100, Aguascalientes, Mexico; gustavobastien14@gmail.com; 4 Facultad de Medicina Campus Xalapa, Unidad de Ciencias de la Salud, Universidad Veracruzana, Médicos y Odontólogos S/N, Unidad del Bosque, Xalapa de Enriquez 91010, Veracruz, Mexico; edreihernandez777@gmail.com (E.L.-H.); vladimirogt1@gmail.com (V.G.-T.); adaircortesgutierrez@gmail.com (A.Z.C.-G.); 5 Facultad de Medicina, Instituto de Ciencias Biomédicas, Universidad Autónoma de Ciudad Juarez, Plutarco Elías Calles 1210, Fraccionamiento Fovissste Chamizal, Ciudad Juárez 32310, Chihuahua, Mexico; fsofia171104@gmail.com; 6 Escuela Nacional de Medicina y Homeopatía, Instituto Politécnico Nacional, Av Guillermo Massieu Helguera 239, La Purísima Ticoman, Gustavo A. Madero, Ciudad de México 0732, Mexico; vargasale19m@gmail.com; 7 Licenciatura en Nutrición, Unidad Académica de Enfermería, Universidad Autónoma de Zacatecas, Carretera Zacatecas-Guadalajara Km. 6, Ejido La Escondida, Zacatecas 98160, Zacatecas, Mexico; charuva@uaz.edu.mx; 8 Facultad de Medicina, Universidad El Bosque, Bogotá 110121, PC, Colombia; natalirodriguez@unbosque.edu.co

**Keywords:** peptic ulcer hemorrhage, proton pump inhibitors, hemostasis, endoscopic, infusions, intravenous, intermittent bolus therapy, rebleeding

## Abstract

**Background/Objectives**: Continuous high-dose intravenous proton pump inhibitor (PPI) infusion is commonly used following endoscopic hemostasis for bleeding peptic ulcers, despite uncertainty regarding its clinical advantage over intermittent administration. This systematic review and meta-analysis evaluated whether continuous infusion improves clinically relevant outcomes compared with intermittent intravenous bolus therapy. **Methods**: A systematic search of PubMed, Scopus, Web of Science, ScienceDirect, and Embase was conducted from inception through to 29 June 2026 to identify randomized controlled trials. Effect estimates were pooled using random-effects models with restricted maximum-likelihood estimation and Hartung–Knapp adjustment. Risk of bias was assessed using RoB 2 and certainty of evidence using GRADE. The protocol was retrospectively registered on OSF. **Results**: Twelve randomized controlled trials involving 1726 participants were included. Across 11 trials (1621 participants), the pooled estimate did not demonstrate a statistically significant reduction in overall rebleeding with continuous infusion compared with intermittent bolus therapy (RR 0.93, 95% CI 0.65–1.35; I^2^ = 1.4%). Likewise, the pooled estimates did not demonstrate superiority of continuous infusion for rebleeding within 24 h (RR 0.70, 95% CI 0.09–5.64), rebleeding within 7 days (RR 1.07, 95% CI 0.66–1.72), surgery (RR 1.42, 95% CI 0.80–2.53), or mortality (RR 1.76, 95% CI 0.90–3.44). Moderate-certainty evidence indicated little or no clinically important difference in blood units transfused or hospital stay. Certainty was very low for rebleeding outcomes and low for surgery and mortality. **Conclusions**: Continuous intravenous PPI infusion was not shown to be superior to intermittent intravenous bolus therapy following successful endoscopic hemostasis. However, the very low- to low-certainty evidence for rebleeding, surgery, and mortality establishes neither equivalence nor noninferiority and does not exclude clinically important benefit or harm. The available trials do not identify the optimal intermittent dose or dosing frequency.

## 1. Introduction

Acute upper gastrointestinal bleeding remains a frequent medical emergency associated with substantial morbidity, hospital resource utilization, and mortality. Although advances in prevention, endoscopic therapy, and pharmacological management have improved clinical outcomes, peptic ulcers remain the most common cause of nonvariceal upper gastrointestinal bleeding [1,2]. Patients with active bleeding, a nonbleeding visible vessel, or an adherent clot are at particularly high risk of recurrent hemorrhage and may require repeat endoscopic treatment, blood transfusion, transcatheter arterial embolization, or surgery. Preventing rebleeding after initial hemostasis is therefore a central objective of post-endoscopic management [1,2].

Current treatment is based on hemodynamic stabilization, early endoscopic evaluation, endoscopic hemostasis for high-risk ulcer stigmata, and subsequent gastric acid suppression with proton pump inhibitors (PPIs) [2]. The therapeutic rationale for intensive acid suppression is supported by experimental evidence showing that acidic conditions and pepsin impair platelet aggregation, coagulation, and clot stability [3]. Consistent with this biological mechanism, PPI therapy following ulcer bleeding reduces recurrent hemorrhage and the need for surgical intervention compared with placebo or histamine-2 receptor antagonists [4]. Consequently, high-dose PPI therapy during the first 72 h after successful endoscopic hemostasis has become an established component of care [5,6].

A previous systematic review and noninferiority meta-analysis found that intermittent PPI therapy was comparable to the conventional bolus-plus-continuous-infusion regimen for preventing recurrent bleeding in patients with endoscopically treated high-risk ulcers [7]. Nevertheless, that review evaluated a broader range of intermittent strategies, including both oral and intravenous regimens, and its literature search was completed in December 2013. Consequently, it did not isolate the evidence derived exclusively from direct randomized comparisons of continuous intravenous infusion versus intermittent intravenous bolus administration, and it preceded subsequently published trials. Furthermore, uncertainty remains regarding less frequent but clinically serious outcomes, including surgery and mortality, as well as transfusion requirements and length of hospital stay. Variation in PPI dose, administration frequency, endoscopic co-interventions, outcome definitions, and follow-up duration further complicates interpretation of the available evidence. Therefore, an updated and methodologically focused synthesis of randomized evidence is warranted to determine whether continuous intravenous infusion provides a clinically meaningful advantage over intermittent intravenous bolus therapy after endoscopic hemostasis. The aim of this systematic review and meta-analysis was to compare the effects of these two administration strategies in patients with bleeding peptic ulcers. The primary outcome was overall ulcer rebleeding during the reported follow-up period. Secondary outcomes included rebleeding within 24 h and 7 days, rebleeding according to gastric or duodenal ulcer location, need for surgical intervention, all-cause mortality, transfusion requirements, and length of hospital stay. Outcome-specific risk-of-bias assessments and GRADE evaluations were incorporated to distinguish absence of statistically significant differences from reliable evidence of comparable clinical effects.

## 2. Materials and Methods

### 2.1. Protocol Registration and Reporting

This systematic review and meta-analysis was conducted and reported in accordance with the Preferred Reporting Items for Systematic Reviews and Meta-Analyses (PRISMA) 2020 Statement [8]. The finalized review protocol was retrospectively registered on the Open Science Framework on 2 August 2026, after completion of the literature search on 29 June 2026, to provide a permanent and transparent public record of the review methods (OSF; registration DOI: https://doi.org/10.17605/OSF.IO/H7XBP). Data extraction, outcome-specific RoB 2 assessment, quantitative synthesis, sensitivity analyses, and GRADE evaluation were conducted after registration. This review compared the efficacy and safety of continuous intravenous infusion versus intermittent intravenous bolus administration of proton pump inhibitors among patients with bleeding peptic ulcers who underwent successful endoscopic hemostasis.

### 2.2. Eligibility Criteria

Studies were selected according to predefined eligibility criteria structured using the Population, Intervention, Comparator, Outcomes, and Study design (PICOS) framework.

Population (P): The target population comprised patients with upper gastrointestinal bleeding caused by peptic ulcers who underwent successful endoscopic hemostasis, without restrictions based on sex or ethnicity. Trials whose randomized populations predominantly matched this clinical framework were eligible. Randomization before endoscopy was not, by itself, an exclusion criterion when high-risk peptic ulcer bleeding was subsequently confirmed endoscopically, the eligible intravenous PPI strategies were directly compared, and most randomized participants underwent endoscopic hemostatic therapy. The influence of any trial that did not fully meet the strict post-hemostasis framework was evaluated in a sensitivity analysis excluding the entire trial. Trials enrolling predominantly adult populations were eligible even when their inclusion criteria permitted adolescents, provided that the study population and clinical management were otherwise consistent with the review question. Studies conducted exclusively in pediatric populations, as well as studies involving non-peptic causes of upper gastrointestinal bleeding, variceal bleeding, malignant ulcers, or experimental animal models, were excluded. Trials predominantly involving patients who did not undergo endoscopic hemostasis were also excluded. Studies enrolling mixed populations were eligible only when data for adults with bleeding peptic ulcers could be extracted separately.

Intervention (I) and comparator (C): Eligible studies directly compared continuous intravenous infusion of a PPI, administered with or without an initial intravenous loading bolus, with intermittent intravenous bolus PPI therapy following endoscopic hemostasis. No restrictions were imposed on the type of PPI, dose, treatment duration, or dosing interval, provided that the study directly compared the two eligible administration strategies. Studies comparing PPIs with placebo, histamine-2 receptor antagonists, endoscopic therapy alone, surgical interventions, or pharmacological treatments other than PPIs were excluded. In multiarm trials, only data from the eligible continuous-infusion and intermittent-bolus groups were considered.

Outcomes (O): The primary efficacy outcome was overall ulcer rebleeding during the longest clinical follow-up period reported by each trial. Additional time-specific analyses evaluated rebleeding within 24 h, within 7 days, and at 30 days, whereas rebleeding within 72 h was considered a prespecified exploratory time-point analysis. Subgroup analyses stratified by ulcer location separately evaluated rebleeding in gastric and duodenal ulcers. Secondary outcomes included the need for surgical intervention, transcatheter arterial embolization, all-cause mortality, receipt of blood transfusion, mean number of blood units transfused, length of hospital stay, and adverse events. Studies reporting at least one eligible outcome were eligible for inclusion in the systematic review. Quantitative synthesis was performed when at least two clinically and methodologically comparable studies reported the same outcome using compatible definitions and follow-up periods.

Study design (S): Only randomized controlled trials (RCTs) directly comparing continuous intravenous PPI infusion with intermittent intravenous bolus PPI therapy were included. Observational studies, cohort studies, case–control studies, case series, case reports, conference abstracts without sufficient outcome data, reviews, editorials, letters, clinical practice guidelines, and animal or in vitro studies were excluded. No restrictions regarding publication year or language were applied during the literature search. However, only reports available as full-text articles were eligible to ensure the complete assessment of study characteristics, methodological quality, and outcome data.

### 2.3. Information Sources and Search Strategy

A comprehensive literature search was conducted on 29 June 2026, in PubMed, Scopus, Web of Science, ScienceDirect, and Embase, with coverage extending from the inception of each electronic source to the search date. The search strategy was developed using controlled vocabulary and free-text terms informed by Medical Subject Headings (MeSH; https://www.ncbi.nlm.nih.gov/mesh/) and Health Sciences Descriptors (DeCS; https://decs.bvsalud.org/en/). The search terms were adapted to the syntax, search fields, and Boolean operators supported by each electronic source while maintaining conceptual equivalence across all searches. No restrictions regarding publication year or language were applied. In addition, the reference lists of the included studies and relevant review articles were manually screened to identify potentially eligible studies not retrieved through the electronic searches. The complete database-specific search strategies, reproduced exactly as executed, together with the corresponding platforms, search fields, search date, limits applied, and numbers of records retrieved, are provided in Supplementary Table S1.

### 2.4. Screening and Eligibility Assessmen

All records identified through the database searches were imported into Mendeley Manager (https://www.mendeley.com/) for reference management and subsequently exported to Rayyan (https://www.rayyan.ai/), where duplicate records were identified and removed. The additional record identified through manual screening of the reference lists of the included studies and relevant review articles was subjected to the same selection process.

Study selection was performed independently by two reviewers according to the predefined eligibility criteria and comprised two sequential phases. In the first phase, the titles and abstracts of all unique records were screened to identify potentially eligible reports. Records considered irrelevant by both reviewers were excluded. In the second phase, the full texts of all reports considered potentially eligible by at least one reviewer were sought and independently assessed against the complete eligibility criteria. Reports for which the full text could not be obtained were recorded as not retrieved and were not subjected to eligibility assessment. Reasons for exclusion at the full-text stage were documented for each report.

Disagreements arising during either phase were resolved through discussion between the two reviewers until consensus was reached. When consensus could not be achieved, a third reviewer was consulted to make the final decision. Multiple reports describing the same randomized trial were linked and treated as reports of a single study to avoid double counting participants. When a secondary or duplicate report did not provide unique eligible information beyond that available in the primary report, it was excluded at the full-text stage; otherwise, all relevant information across linked reports was considered collectively.

The study selection process was documented using the PRISMA 2020 flow diagram, including the numbers of records identified, duplicate records removed, unique records screened, reports sought for retrieval, reports not retrieved, full-text reports assessed for eligibility, reports excluded with reasons, and studies ultimately included in the systematic review and quantitative synthesis.

### 2.5. Data Extraction

Data extraction was performed independently by two reviewers using a standardized data extraction form developed specifically for this review. Extracted information included bibliographic characteristics (first author and year of publication); study characteristics (country, study design, sample size, participant characteristics, endoscopic hemostatic therapy, and duration of follow-up); intervention characteristics (type of PPI, loading dose, maintenance dose, route of administration, treatment duration, and post-endoscopic management); and data for all eligible outcomes.

Outcome data were extracted according to the outcome framework established for the review. The primary efficacy outcome was ulcer rebleeding, evaluated separately as overall rebleeding during the longest follow-up period reported by each study, rebleeding within 24 h, and rebleeding within 7 days. Rebleeding within 72 h was extracted and evaluated as an exploratory outcome. Data on rebleeding according to ulcer location (gastric or duodenal) were extracted for subgroup analyses of the primary efficacy outcome. Secondary outcomes included the need for surgical intervention, transcatheter arterial embolization, all-cause mortality, the number of patients requiring blood transfusion, the mean number of blood units transfused, and length of hospital stay.

For studies included in the quantitative synthesis, dichotomous outcome data were extracted as the number of events and the total number of participants in each treatment group, whereas continuous outcome data were extracted as means, standard deviations, and sample sizes whenever available. Data were obtained directly from the numerical information reported in the text, tables, and Supplementary Materials of the original publications.

For the exploratory 72-h analysis, event counts explicitly reported for rebleeding within 72 h or within the first three days were extracted. When a separate cumulative count was not provided but the timing of individual rebleeding events was reported, events were classified according to whether they occurred within the first 72 h; no interpolation, proportional allocation, or imputation was used. When a trial included multiple eligible continuous-infusion arms sharing a single intermittent comparator, dichotomous data were combined by summing the events and participants across the continuous-infusion arms for each applicable time point, and the common comparator was included only once. No eligible continuous outcome from a multi-arm trial contributed to the quantitative syntheses; therefore, no means or standard deviations were combined. If time-specific counts were internally inconsistent across different parts of a report, the explicitly labeled 72-h counts that were compatible with the reported overall event totals were retained, and robustness was examined in a sensitivity analysis excluding the affected trial.

When multiple publications reported data from the same trial, the reports were linked and treated as a single study, and participants were counted only once. When continuous outcome values reported in a primary publication were internally inconsistent and could not be reliably interpreted, the corresponding mean and standard deviation were extracted from a complementary publication reporting the same cohort and treatment groups. The complementary publication was used exclusively as the data source for the relevant outcome and was not considered an independent study.

Any discrepancies in data extraction were resolved through discussion and consensus between the two reviewers. Before quantitative synthesis, the extracted data were independently verified against the original publications to ensure the accuracy, completeness, and consistency of the final dataset.

### 2.6. Risk of Bias Assessment

The risk of bias in the included randomized controlled trials was assessed independently by two reviewers using the revised Cochrane risk-of-bias tool for randomized trials (RoB 2; version 22 August 2019) [9]. Because RoB 2 judgments are specific to individual trial results rather than to studies as a whole, separate assessments were performed for each trial result contributing to the following outcomes: overall ulcer rebleeding at the longest clinical follow-up reported by each trial, rebleeding within 24 h, rebleeding within 7 days, need for surgical intervention, all-cause mortality, mean number of blood units transfused, and length of hospital stay. The effect of interest was the effect of assignment to the intervention at randomization, consistent with the intention-to-treat principle. For the main graphical summary, risk-of-bias judgments were presented for the primary outcome, overall ulcer rebleeding at the longest follow-up reported by each trial, whereas the complete outcome-specific assessments are provided in Supplementary Table S2. Any disagreements between the reviewers were resolved through discussion and consensus.

Five domains were evaluated: bias arising from the randomization process; bias due to deviations from the intended interventions; bias due to missing outcome data; bias in the measurement of the outcome; and bias in the selection of the reported result. Signaling questions were answered as “yes,” “probably yes,” “probably no,” “no,” “no information,” or “not applicable,” as appropriate. The RoB 2 algorithms were used to inform the domain-level judgments, which were categorized as low risk of bias, some concerns, or high risk of bias.

The overall risk-of-bias judgment for each assessed result was classified as low when all domains were judged to be at low risk; as some concerns when at least one domain raised some concerns but none was judged to be at high risk; and as high when at least one domain was judged to be at high risk or when multiple domains raised concerns that substantially reduced confidence in the result. Disagreements between reviewers were resolved through discussion and consensus. No numerical summary score was calculated.

### 2.7. Quantitative Synthesis and Statistical Analysis

Quantitative synthesis was performed when at least two studies reported clinically and methodologically comparable data for the same outcome. For time-specific analyses, studies were pooled only when they reported the same or a sufficiently comparable follow-up period. The individual randomized participant was considered the unit of analysis, and only one effect estimate from each trial was included in any given meta-analysis to avoid double counting. Continuous intravenous PPI infusion was defined as the experimental intervention and intermittent intravenous bolus therapy as the comparator.

The primary efficacy outcome was study-defined overall clinical ulcer rebleeding during the longest clinical follow-up period reported by each trial. The longest follow-up was selected using a rule applied uniformly across trials and without reference to the magnitude or direction of the observed treatment effect; each trial therefore contributed only one result to this synthesis. This approach maximized the inclusion of eligible trials reporting clinically defined rebleeding; however, because follow-up duration varied, the resulting pooled estimate represents study-defined overall rebleeding rather than rebleeding at a common fixed time point. Prespecified time-specific analyses evaluated rebleeding within 24 h, 7 days, and 30 days, whereas rebleeding within 72 h was considered a prespecified exploratory time-point analysis. The 30-day analysis was restricted to trials reporting rebleeding at that assessment point and was used to examine the robustness of the findings to differences in follow-up duration. Rebleeding in gastric and duodenal ulcers was evaluated separately in prespecified subgroup analyses stratified by ulcer location. Secondary outcomes included the need for surgical intervention, transcatheter arterial embolization, all-cause mortality, receipt of blood transfusion, mean number of blood units transfused, length of hospital stay, and adverse events.

For dichotomous outcomes, treatment effects were expressed as risk ratios with 95% CIs. Random-effects estimates were pooled using inverse-variance weighting. An RR below 1.0 indicated a lower risk of the corresponding outcome with continuous infusion. Continuous outcomes reported using the same unit of measurement were summarized as mean differences (MDs) with 95% CIs using the inverse-variance method [10]. Receipt of blood transfusion was analyzed as a dichotomous outcome using the RR, whereas the mean number of blood units transfused was analyzed as a continuous outcome using the MD; these transfusion-related outcomes were therefore synthesized separately. Length of hospital stay was pooled only when reported as mean days with an available standard deviation. Outcomes reported using non-comparable metrics were not statistically pooled. Medians and ranges were not converted into means and standard deviations and were retained for narrative synthesis.

Random-effects models were used because clinical and methodological variability was anticipated across trials, including differences in PPI regimens, participant characteristics, endoscopic management, co-interventions, and follow-up duration. Between-study variance (τ^2^) was estimated using the restricted maximum-likelihood method [10]. The Hartung–Knapp method was used to calculate 95% CIs for the pooled random-effects estimates [10,11].

For dichotomous outcomes containing a zero cell in a study-level 2 × 2 table, a continuity correction of 0.5 was applied, according to the implementation in the meta package, to permit calculation of the study-specific RR and its standard error. Trials with zero events in both treatment groups were retained in the descriptive presentation and participant totals but did not contribute to the pooled RR because they provided no information regarding the direction or magnitude of the relative treatment effect [10,12].

To assess the robustness of outcomes affected by zero-cell handling, additional post hoc common-effect Mantel–Haenszel risk-difference analyses were performed using the original, uncorrected cell counts. These analyses were applied to all outcomes whose primary risk-ratio synthesis contained at least one zero cell and retained trials with zero events in both treatment groups. Risk differences were calculated as the risk in the continuous-infusion group minus the risk in the intermittent-bolus group and are reported as absolute percentage-point differences [10,12].

Statistical heterogeneity was assessed using Cochran’s Q test, with *p* < 0.10 indicating evidence of heterogeneity, and quantified using τ^2^ and the I^2^ statistic. I^2^ values were interpreted cautiously as approximately 0–40%, potentially unimportant; 30–60%, moderate; 50–90%, substantial; and 75–100%, considerable heterogeneity. These thresholds were used as general interpretative guidance rather than rigid decision rules [10].

The registered protocol prespecified sensitivity analyses for the primary overall-rebleeding outcome excluding trials in which one treatment group received an additional co-intervention not applied to the other group, such as scheduled second-look endoscopy with retreatment, and excluding trials judged to be at high overall risk of bias. A combined analysis applying both exclusions was also prespecified. A common-effect Mantel–Haenszel analysis of the primary overall-rebleeding outcome was likewise prespecified. After registration, the co-intervention sensitivity analysis was extended to other applicable outcomes, and the common-effect analyses were extended to rebleeding within 24 h, 7 days, and the exploratory 72-h assessment. Additional post-registration sensitivity analyses excluded trials that did not strictly conform to the post-hemostasis framework, defined as trials in which randomization occurred before endoscopy or endoscopic hemostatic therapy was not administered to all randomized participants, and excluded the trial with internally inconsistent 72-h event counts. These post-registration analyses were performed only when at least two studies remained after exclusion.

Small-study effects were assessed only for outcomes with at least 10 studies contributing estimable effect sizes. Contour-enhanced funnel plots were inspected visually, and funnel plot asymmetry was evaluated using Egger’s linear regression test. Accordingly, these assessments were performed for overall clinical ulcer rebleeding and the need for surgery, which included 11 and 10 informative studies, respectively. Small-study effects were not formally assessed for all-cause mortality because only eight studies contributed estimable effect sizes. Funnel plot asymmetry was interpreted as evidence of potential small-study effects and not as definitive evidence of publication bias, because asymmetry may also arise from sparse events, clinical or methodological heterogeneity, or chance [10,12].

Tests of pooled treatment effects and funnel plot asymmetry were two-sided, with *p* < 0.05 considered statistically significant, whereas Cochran’s Q test was evaluated using *p* < 0.10. Statistical analyses were performed in R using the meta package, version 8.5-0, and results were presented using forest plots and, where appropriate, contour-enhanced funnel plots [12].

The primary outcome definition, effect measures, and primary statistical model were not changed after registration, and no prespecified outcome was removed or redefined. All methodological additions made after registration were limited to the sensitivity analyses described above and were explicitly identified as post-registration or post hoc analyses.

### 2.8. Assessment of the Certainty of Evidence

The certainty of the evidence was assessed for each critical or important outcome included in the Summary of Findings (SoF) table using the Grading of Recommendations Assessment, Development and Evaluation (GRADE) approach [13,14]. Assessments were conducted independently by two reviewers, and disagreements were resolved through discussion and consensus. Because the evidence was derived exclusively from RCTs, the certainty of each body of evidence was initially rated as high. Certainty was downgraded by one level for serious concerns or by two levels for very serious concerns across five domains: risk of bias, inconsistency, indirectness, imprecision, and publication bias. The resulting certainty of evidence was classified as high, moderate, low, or very low [13,14].

Risk-of-bias judgments were informed by the outcome-specific assessments conducted using the revised Cochrane risk-of-bias tool for randomized trials (RoB 2), considering both the judgments for the individual trials and their relative contributions to the corresponding pooled effect estimate. Inconsistency was evaluated by examining the direction and magnitude of study-specific effects, overlap of CIs, between-study variance (τ^2^), the I^2^ statistic, and plausible clinical or methodological explanations for any observed variability. Statistical measures of heterogeneity were not used as the sole basis for downgrading. Indirectness was assessed according to the applicability of the available evidence to the review question in terms of population, intervention, comparator, outcome measurement, and follow-up period.

Imprecision was assessed using a minimally contextualized approach. For dichotomous outcomes, risk ratios of 0.75 and 1.25 were used as thresholds to define a clinically important benefit and harm, respectively. For continuous outcomes, mean differences of 1 unit of blood and 1 day of hospital stay in either direction were considered clinically important. The 95% CI was analyzed in relation to the null effect and these predefined thresholds, while also considering the total number of participants and events, as well as whether the optimal information size (OIS) had been reached [13,15]. The certainty level was downgraded when the CI included clinically important effects leading to substantially different clinical conclusions, or when the available information was insufficient to provide a reliable estimate of the effect.

Publication bias was assessed for each outcome by considering the comprehensiveness of the search, evidence of selective non-reporting, availability of trial registrations or protocols, study sponsorship, and the distribution of study sizes. For outcomes with at least 10 studies contributing estimable effect sizes, small-study effects were additionally assessed through visual inspection of contour-enhanced funnel plots and Egger’s linear regression test. Accordingly, these analyses were performed for overall clinical ulcer rebleeding and the need for surgery. Funnel plot asymmetry was interpreted as evidence of potential small-study effects rather than definitive evidence of publication bias. Statistical assessment was not performed for outcomes with fewer than 10 informative studies because of the limited power of asymmetry tests under these conditions [10].

A Summary of Findings table was prepared for the comparison between continuous intravenous PPI infusion and intermittent intravenous PPI therapy. The table included seven clinically important outcomes: overall ulcer rebleeding during the longest clinical follow-up period reported by each trial; ulcer rebleeding within 24 h; ulcer rebleeding within 7 days; need for surgical intervention; all-cause mortality; mean number of blood units transfused; and length of hospital stay. Rebleeding within 72 h and analyses stratified by gastric or duodenal ulcer location were not included because they represented an exploratory time-point analysis and subgroup analyses, respectively. The study-defined dichotomous transfusion outcome was excluded because definitions differed across trials, whereas transcatheter arterial embolization was excluded because fewer than two trials reported comparable data. Adverse events likewise remained a prespecified secondary outcome but were not quantitatively synthesized because their reporting was incomplete and insufficiently comparable across trials.

For dichotomous outcomes, relative effects were presented as risk ratios with 95% CIs, together with anticipated absolute effects based on the risk observed in the intermittent-therapy group. For continuous outcomes, effects were presented as mean differences with 95% CIs. All decisions to downgrade the certainty of evidence were explicitly documented and justified in explanatory footnotes accompanying the SoF table [13,14].

## 3. Results

### 3.1. Study Selection

The systematic literature search identified 6572 records across five electronic databases: PubMed (n = 388), Scopus (n = 2030), Web of Science (n = 420), ScienceDirect (n = 2230), and Embase (n = 1504). After the removal of 1615 duplicate records, 4957 unique records remained for title and abstract screening. Of these, 4944 were excluded because they did not meet the predefined eligibility criteria, leaving 13 reports for full-text assessment. Manual screening of the reference lists of eligible studies and relevant review articles identified one additional potentially eligible report that had not been retrieved through the electronic database searches. Consequently, 14 full-text reports were assessed for eligibility. One report was excluded because it evaluated patients with artificial ulcers following endoscopic submucosal dissection, which did not meet the predefined population criteria. Among the 13 eligible reports, two publications described the same randomized trial and study population. These publications were linked and treated as multiple reports of a single study [16,17,18,19,20,21,22,23,24,25,26,27].

The complementary publication by Mehmedović-Redžepović et al. [28] was used only as an additional source of relevant outcome data for the trial reported by Mesihović et al. [22], and the participants were counted only once. Therefore, 13 reports corresponding to 12 unique randomized controlled trials were included in the systematic review. All 12 trials contributed data to at least one quantitative synthesis. The complete study selection process is presented in the PRISMA 2020 flow diagram (Figure 1).

### 3.2. Characteristics of the Included Studies

The 12 included RCTs were published between 2007 and 2024 and were conducted in 10 countries or regions across Asia and Europe (Table 1A) [16,17,18,19,20,21,22,23,24,25,26,27]. Two trials were conducted in Hong Kong [16,18], two in Taiwan [24,27], and one each in Spain [17], Italy [20], Turkey [21], Bosnia and Herzegovina [22], South Korea [23], Singapore [25], Thailand [26], and Iran [19]. Eleven trials were conducted at a single center, whereas Andriulli et al. [20] performed the only multicenter study, involving 11 Italian centers. Hung et al. [16] and Choi et al. [23] used three-arm designs, Chan et al. [25] conducted an equivalence trial, and Chiu et al. [18] used a noninferiority design.

Across the eligible continuous-infusion and intermittent-bolus arms, 1726 participants were randomized: 876 to continuous intravenous PPI infusion and 850 to intermittent intravenous bolus therapy (Table 1A). Individual randomized sample sizes ranged from 28 participants in Yamada and Wongwanakul [26] to 482 in Andriulli et al. [20]. After the post-randomization exclusions reported by Hung et al. [16], Andriulli et al. [20], Yüksel et al. [21], and Chan et al. [25], 1701 participants were included in the principal published analyses. Hung et al. [16] additionally included a no-treatment group, which was not eligible for the present comparison. Choi et al. [23] evaluated two continuous-infusion regimens against a single intermittent-bolus group; the two continuous arms were therefore represented together for study-level characterization without duplicating the comparator group.

All trials evaluated patients with upper gastrointestinal bleeding caused by peptic ulcers with high-risk endoscopic stigmata (Table 1B). In most studies, randomization was performed after successful endoscopic hemostasis. Garrido et al. [17] randomized patients at admission, and endoscopic therapy was subsequently administered to 90% of the continuous-infusion group and all participants in the intermittent-bolus group. This trial was retained because it directly compared the eligible intravenous PPI regimens in patients with endoscopically confirmed high-risk peptic ulcer bleeding. However, because randomization preceded endoscopy and not all participants underwent endoscopic hemostatic therapy, it was excluded from the strict post-hemostasis sensitivity analysis. Eight trials included Forrest Ia, Ib, IIa, and IIb lesions [16,18,20,22,23,24,25,26]. Garrido et al. [17], Yüksel et al. [21], and Chen et al. [27] included Forrest Ia, Ib, and IIa lesions, whereas Jafari et al. [19] evaluated patients with nonbleeding visible vessels or adherent clots, corresponding to Forrest IIa and IIb, respectively. Gastric and duodenal ulcers were represented in all trials reporting ulcer location. Chan et al. [25] also included stomal ulcers, Chiu et al. [18] included anastomotic ulcers, and Jafari et al. [19] did not report ulcer location.

The reported mean ages ranged from 52.87 to 67.1 years in the continuous-infusion groups and from 48.87 to 67.4 years in the intermittent-bolus groups (Table 1A). Although Choi et al. [23] and Chen et al. [27] permitted enrollment from 16 years of age and Chiu et al. [18] included patients aged 15–90 years, the reported mean ages indicated predominantly adult populations. None of these studies separately reported the number or outcomes of participants younger than 18 years.

Endoscopic management generally consisted of diluted epinephrine or adrenaline injection, either alone or combined with thermal or mechanical hemostatic techniques (Table 1B). Thermal modalities included heater-probe coagulation, bipolar or multipolar electrocoagulation, and argon plasma coagulation, whereas mechanical therapy primarily involved hemoclip placement. Garrido et al. [17] used epinephrine combined with polidocanol and performed routine endoscopic reassessment at 24–48 h. Chiu et al. [18] incorporated scheduled second-look endoscopy exclusively into the intermittent-bolus comparator strategy. Pantoprazole was the only PPI evaluated in nine trials [16,17,19,21,22,23,24,26,27], omeprazole was used in two trials [18,25], and Andriulli et al. [20] permitted either omeprazole or pantoprazole (Table 1B).

### 3.3. Intervention Characteristics and Reported Outcomes

The intervention characteristics and reported outcomes of the 12 included randomized controlled trials are summarized in Table 2. All trials compared continuous intravenous PPI infusion with an intermittent intravenous PPI regimen in patients with bleeding peptic ulcers undergoing endoscopic management [16,17,18,19,20,21,22,23,24,25,26,27]. In 11 trials, randomization occurred after successful endoscopic hemostasis [16,18,19,20,21,22,23,24,25,26,27]. In contrast, Garrido et al. [17] randomized patients at admission; endoscopic therapy was subsequently administered to 90% of the continuous-infusion group and 100% of the intermittent group. The high-dose continuous regimen consisting of an 80-mg intravenous loading dose followed by infusion at 8 mg/h for 72 h was used in 10 trials [16,17,18,20,21,23,24,25,26,27]. Choi et al. [23] additionally evaluated a lower-dose continuous regimen comprising a 40-mg loading dose followed by infusion at 4 mg/h for 72 h. Mesihović et al. [22] and Jafari et al. [19] administered continuous pantoprazole at 8 mg/h for three days but did not clearly report a separate loading dose. In Yamada and Wongwanakul [26], the 80-mg loading dose was administered to all participants before endoscopic treatment and randomization; the continuous-infusion group subsequently received 8 mg/h on days 1–3 and intermittent pantoprazole from days 4 to 7.

The intermittent regimens differed substantially in dosing interval and total daily dose. Pantoprazole or omeprazole 40 mg once daily was used in five trials [17,20,23,25,27], whereas 40 mg every 12 h was administered in five trials [16,18,21,22,26]. Hsu et al. [24] used pantoprazole 40 mg every 6 h after a common 80-mg loading dose, while Jafari et al. [19] administered pantoprazole 40 mg every 8 h. Most intravenous comparisons lasted 72 h; Yamada and Wongwanakul [26] extended intravenous treatment to seven days. Several studies subsequently transitioned both groups to a common oral acid-suppressive regimen [16,18,20,22,23,24,25,26,27].

Post-endoscopic management also varied among trials. Repeat endoscopy was generally performed when recurrent bleeding was clinically suspected. Garrido et al. [17] conducted routine endoscopic reassessment at 24–48 h in both groups, whereas Jafari et al. [19] performed scheduled upper endoscopy on day 3 in all participants to evaluate changes in bleeding stigmata. Andriulli et al. [20] and Mesihović et al. [22] selectively repeated endoscopy in patients with a Rockall score ≥6 and required emergency endoscopy when rebleeding was suspected. In Chiu et al. [18], scheduled second-look endoscopy at 16–24 h and retreatment of persistent high-risk stigmata were incorporated exclusively into the intermittent comparator strategy. Therefore, the comparator in that trial represented a combined pharmacological and procedural intervention.

The duration of primary clinical outcome assessment ranged from 72 h in Yüksel et al. [21] and Jafari et al. [19] to seven days in Yamada and Wongwanakul [26], 14 days in Hsu et al. [24], and 30 days in Hung et al. [16], Choi et al. [23], Chan et al. [25], Chen et al. [27], and Chiu et al. [18]. Garrido et al. [17], Andriulli et al. [20], and Mesihović et al. [22] primarily reported in-hospital outcomes. Choi et al. [23] additionally evaluated 24-h intragastric pH and pharmacokinetic parameters, while Hung et al. [16] assessed intragastric pH and performed follow-up endoscopy to document ulcer healing. Definitions of rebleeding were not uniform across trials. Most studies evaluated clinically apparent recurrent bleeding using one or more criteria such as recurrent hematemesis or melena, hemodynamic deterioration, a decrease in hemoglobin, renewed transfusion requirements, or endoscopic confirmation. However, the required combination of criteria and the use of confirmatory endoscopy varied, and some trials combined persistent and recurrent bleeding [16,17,18,19,20,21,22,23,24,25,26,27]. Jafari et al. [19] instead defined treatment failure using scheduled day-3 endoscopic evidence of persistent or recurrent high-risk bleeding stigmata rather than clinically detected rebleeding; therefore, this trial was excluded from the primary clinical-rebleeding synthesis. Surgical intervention and mortality were reported in 11 trials [16,17,18,19,20,21,22,24,25,26,27], as were transfusion-related data [16,17,18,19,20,21,22,24,25,26,27]. Length of hospital stay was available from 10 trials [16,18,19,20,21,22,24,25,26,27]. Overall, the principal sources of clinical heterogeneity were the reporting and timing of loading doses, intermittent dosing intervals, duration of intravenous therapy, transition to oral treatment, outcome-assessment periods, and use of scheduled second-look endoscopy. These differences should be considered when interpreting the subsequent quantitative syntheses (Table 2B).

### 3.4. Quantitative Data Available for Evidence Synthesis

The quantitative data available for the primary outcome, time-specific analyses, exploratory analyses, subgroup analyses, and secondary outcomes are summarized in Table 3. Across the eligible comparison arms, the 12 randomized controlled trials randomized 1726 participants: 876 to continuous intravenous PPI infusion and 850 to an intermittent intravenous regimen [16,17,18,19,20,21,22,23,24,25,26,27]. All 12 trials contributed data to at least one quantitative synthesis, although outcome-specific sample sizes were smaller because of differences in outcome availability and the analysis populations reported by the individual studies (Table 3A).

Clinical ulcer rebleeding data were available from 11 trials comprising 1621 participants, of whom 823 received continuous infusion and 798 received intermittent therapy [16,17,18,20,21,22,23,24,25,26,27]. This constituted the primary efficacy synthesis and used the principal clinical follow-up period reported by each trial, ranging from 72 h or the index hospitalization to 30 days. Choi et al. [23] evaluated two continuous-infusion regimens against a single intermittent comparator. For each applicable dichotomous rebleeding analysis, the events and participants from the two continuous-infusion arms were summed, whereas the common intermittent comparator was included only once. No eligible continuous outcome from this trial contributed to a quantitative synthesis; consequently, no means or standard deviations were combined. Jafari et al. [19] assessed treatment failure based on the scheduled day-3 endoscopic appearance rather than an equivalent clinical definition of recurrent bleeding and was therefore excluded from the primary clinical-rebleeding synthesis. Data for the time-specific analyses were available from fewer trials. Rebleeding within 24 h was reported by Andriulli et al. [20], Mesihović et al. [22], and Chiu et al. [18], providing data from 848 participants. Six trials comprising 1138 participants provided or permitted the unambiguous extraction of rebleeding within seven days [18,20,22,23,26,27].

Rebleeding within 72 h was evaluated as an exploratory time-point analysis. Five trials comprising 861 participants contributed data: 441 assigned to continuous infusion and 420 to intermittent therapy [20,21,23,26,27]. Rebleeding occurred in 31 of 441 participants receiving continuous infusion and in 32 of 420 receiving intermittent therapy. Yüksel et al. [21], Yamada and Wongwanakul [26], and Chen et al. [27] directly reported rebleeding within 72 h or the first three days. In Choi et al. [23], the reported timing of the individual events permitted unambiguous classification: all three events in the intermittent group occurred within 72 h, whereas the only event in either continuous-infusion arm occurred on day 6. The two continuous-infusion arms were therefore combined as 0/40 for this analysis, and the common intermittent comparator was included only once. For Andriulli et al. [20], the abstract-reported 72-h counts of 18/238 and 19/236 were used; because these differed from the three-day counts reported in the main text and the latter were incompatible with the total in-hospital counts in Table 1, the robustness of the result was examined by excluding this trial. Mesihović et al. [22] was not included in this analysis because events reported between >24 h and 7 days could not be classified according to whether they occurred before or after 72 h.

Secondary clinical outcomes were reported with varying completeness. Data on the need for surgery and all-cause mortality were available from 11 trials [16,17,18,19,20,21,22,24,25,26,27], contributing 1632 and 1634 participants, respectively. The slight difference in sample size resulted from the outcome-specific analysis populations reported by Yüksel et al. [21]. Double-zero trials were retained when documenting data availability, although they did not contribute statistical weight to the corresponding risk-ratio calculation. Transcatheter arterial embolization remained a prespecified secondary outcome but was not quantitatively synthesized because fewer than two trials reported comparable data.

Five trials comprising 761 participants reported a dichotomous transfusion outcome (Table 3A) [17,19,20,21,22]. However, its definition was not uniform: Garrido et al. [17], Yüksel et al. [21], and Jafari et al. [19] reported the number of patients receiving any transfusion, whereas Andriulli et al. [20] and Mesihović et al. [22] reported the number receiving at least two units. These study-defined outcomes were therefore combined in an exploratory synthesis and interpreted cautiously. Continuous transfusion requirements expressed as mean blood units with standard deviations were available from seven trials comprising 1288 participants [18,20,21,22,25,26,27]. For the Mesihović trial, the mean and standard deviation were obtained from the complementary publication by Mehmedović-Redžepović et al. [28], which reported the same cohort and treatment groups; the trial and its participants were counted only once. Hung et al. [16] reported mean transfusion requirements without standard deviations, whereas Hsu et al. [24] reported transfusion volume in milliliters without standard deviations suitable for conversion. These data were therefore not included in the continuous synthesis.

Mean length of hospital stay with corresponding standard deviations was available from seven trials comprising 1063 participants [19,20,21,22,25,26,27]. Hung et al. [16] and Hsu et al. [24] reported mean hospital stay without standard deviations, whereas Chiu et al. [18] reported this outcome as median and range. Consequently, these three trials were not included in the corresponding quantitative synthesis.

Overall, 11 quantitative syntheses were supported by the updated dataset: one primary analysis of overall clinical ulcer rebleeding; three time-specific analyses examining rebleeding within 24 h, within seven days, and within 72 h, with the latter considered exploratory; two subgroup analyses according to gastric or duodenal ulcer location; and five secondary analyses evaluating the need for surgery, all-cause mortality, the study-defined dichotomous transfusion outcome, mean blood units transfused, and length of hospital stay. Depending on outcome availability, between three and 11 randomized controlled trials contributed to each synthesis.

### 3.5. Risk of Bias in Included Studies

Risk of bias was assessed for each reported outcome in the 12 included randomized controlled trials [16,17,18,19,20,21,22,23,24,25,26,27]. In accordance with the outcome-specific framework of RoB 2, Figure 2 presents the assessment for the primary outcome, overall ulcer rebleeding at the longest follow-up reported by each trial. Complete judgments for all secondary outcomes are provided in Supplementary Table S2. Eleven trials contributed an evaluable result for the primary outcome [16,17,18,20,21,22,23,24,25,26,27]. Jafari et al. [19] did not report a clinically defined rebleeding outcome compatible with the primary outcome and is therefore classified as not applicable in this figure.

For overall ulcer rebleeding, one of the 11 evaluable trials (9.1%) was judged to have an overall low risk of bias, eight (72.7%) raised some concerns, and two (18.2%) were classified as having a high risk of bias. Chan et al. [25] was the only trial judged to be at low risk across all five domains. Hung et al. [16] and Yüksel et al. [21] were classified as having a high overall risk of bias, primarily because of concerns related to deviations from the intended interventions and, in the Hung trial, missing outcome data.

Regarding individual domains, four trials (36.4%) were judged to be at low risk for the randomization process, whereas seven (63.6%) raised some concerns because sequence generation or allocation concealment was insufficiently described. Nine trials (81.8%) were at low risk for deviations from the intended interventions, while Hung et al. [16] and Yüksel et al. [21] were judged to be at high risk in this domain. Missing outcome data were judged to be at low risk in nine trials (81.8%), with some concerns in Yüksel et al. [21] and high risk in Hung et al. [16]. Concerns were more frequent for outcome measurement and selection of the reported result: eight trials (72.7%) raised some concerns regarding outcome measurement, and ten (90.9%) raised some concerns regarding selection of the reported result, mainly because prospective protocols or sufficiently detailed statistical analysis plans were unavailable.

Across the complete outcome-specific assessment, Hung et al. [16] remained at high risk for all evaluable outcomes. Yüksel et al. [21] was judged to be at high risk for rebleeding and surgical intervention but raised some concerns for the remaining outcomes. Chan et al. [25] was at low risk for overall rebleeding, surgery, mortality, and length of hospital stay, with some concerns only for the mean number of blood units transfused. The remaining nine trials raised some concerns for all their evaluable outcomes. No outcome was classified as having a high overall risk of bias solely because of bias in selection of the reported result.

### 3.6. Meta-Analysis of Clinical Outcomes

Meta-analyses were conducted for clinical outcomes reported by at least two randomized controlled trials with sufficiently comparable definitions and available numerical data. Dichotomous outcomes were summarized using risk ratios (RRs), whereas continuous outcomes were analyzed using mean differences (MDs), each with 95% CIs. Random-effects models were fitted using restricted maximum-likelihood estimation, with the Hartung–Knapp adjustment applied to the CIs. The findings are presented below according to the timing and type of outcome evaluated.

#### 3.6.1. Overall Ulcer Rebleeding

Of the 12 included randomized controlled trials, 11 provided clinically defined data on overall ulcer rebleeding at the longest reported follow-up and were included in the meta-analysis (Figure 3).

These trials comprised 1621 participants and 148 rebleeding events. Rebleeding occurred in 72 of 823 patients receiving continuous PPI infusion and in 76 of 798 patients receiving intermittent bolus therapy. The random-effects meta-analysis showed no statistically significant difference between treatment strategies (RR = 0.93; 95% CI: 0.65–1.35; *p* = 0.691). Between-study heterogeneity was low (I^2^ = 1.4%; τ^2^ = 0.029; Q = 10.14, *p* = 0.428). The 95% prediction interval ranged from 0.54 to 1.61, indicating that the underlying effect in a future comparable trial could plausibly favor either treatment strategy. Jafari et al. [19] was not included because it did not report a clinically defined rebleeding outcome compatible with this analysis.

#### 3.6.2. Ulcer Rebleeding Within 24 Hours

Three randomized controlled trials comprising 848 participants evaluated clinical ulcer rebleeding within 24 h (Figure 4). Rebleeding occurred in 16 of 425 patients receiving continuous PPI infusion and in 19 of 423 patients receiving intermittent bolus therapy. The random-effects meta-analysis showed no statistically significant difference between treatment strategies (RR = 0.70; 95% CI: 0.09–5.64; *p* = 0.538). Between-study heterogeneity was moderate but imprecisely estimated (I^2^ = 34.6%; τ^2^ = 0.343; Q = 3.06, *p* = 0.217). Although the point estimate favored continuous infusion, the wide CI indicated substantial uncertainty regarding the direction and magnitude of the treatment effect.

#### 3.6.3. Ulcer Rebleeding Within 7 Days

Six randomized controlled trials comprising 1138 participants evaluated clinical ulcer rebleeding within seven days (Figure 5). Rebleeding occurred in 55 of 580 patients receiving continuous PPI infusion and in 50 of 558 patients receiving intermittent bolus therapy. The random-effects meta-analysis showed no statistically significant difference between treatment strategies (RR = 1.07; 95% CI: 0.66–1.72; *p* = 0.743). No between-study heterogeneity was observed based on the point estimate (I^2^ = 0%; τ^2^ = 0.003; Q = 4.98, *p* = 0.418), although the CI for I^2^ was wide (0–74.6%). Overall, the available evidence did not demonstrate that either administration strategy reduced the risk of ulcer rebleeding within seven days.

#### 3.6.4. Exploratory Analysis of Ulcer Rebleeding Within 72 Hours

Five randomized controlled trials comprising 861 participants contributed data to the exploratory analysis of clinical ulcer rebleeding within 72 h (Figure 6). Rebleeding occurred in 31 of 441 participants receiving continuous PPI infusion and in 32 of 420 participants receiving intermittent bolus therapy. The random-effects meta-analysis showed no statistically significant difference between treatment strategies (RR = 0.96; 95% CI: 0.46–2.04; *p* = 0.897). Between-study heterogeneity was low based on the point estimate (I^2^ = 14.5%; τ^2^ < 0.0001; Q = 4.68, *p* = 0.322), although the CI for I^2^ was wide (0–82.2%). The pooled estimate was close to the null effect; however, the wide CI indicated substantial uncertainty regarding the relative effects of the two administration strategies.

#### 3.6.5. Clinical Ulcer Rebleeding in Gastric Ulcers

Three randomized controlled trials comprising 255 participants contributed data to the exploratory subgroup analysis of clinical rebleeding in gastric ulcers (Figure 7). Rebleeding occurred in 18 of 124 participants receiving continuous PPI infusion and in 17 of 131 participants receiving intermittent bolus therapy. The random-effects meta-analysis showed no statistically significant difference between treatment strategies (RR = 1.11; 95% CI: 0.34–3.61; *p* = 0.737). No between-study heterogeneity was observed based on the point estimates (I^2^ = 0%; τ^2^ = 0; Q = 1.49, *p* = 0.474), although the CI for I^2^ was wide (0–89.6%). The wide CI around the pooled effect indicated substantial uncertainty and did not support the superiority of either administration strategy for gastric-ulcer rebleeding.

#### 3.6.6. Clinical Ulcer Rebleeding in Duodenal Ulcers

Three randomized controlled trials comprising 314 participants contributed data to the exploratory subgroup analysis of clinical rebleeding in duodenal ulcers (Figure 8). Rebleeding occurred in 20 of 162 participants receiving continuous PPI infusion and in 14 of 152 participants receiving intermittent bolus therapy. The random-effects meta-analysis showed no statistically significant difference between treatment strategies (RR = 1.00; 95% CI: 0.28–3.55; *p* = 0.997). Between-study heterogeneity was low based on the point estimate (I^2^ = 5.3%; τ^2^ = 0.094; Q = 2.11, *p* = 0.348), although the CI for I^2^ was wide (0–90.2%). The pooled estimate was centered on the null effect, but the wide CI indicated substantial uncertainty and did not support the superiority of either administration strategy for duodenal-ulcer rebleeding.

#### 3.6.7. Need for Surgery

Eleven randomized controlled trials comprising 1632 participants reported the need for surgery (Figure 9). Surgery was required in 24 of 820 participants receiving continuous PPI infusion and in 16 of 812 participants receiving intermittent bolus therapy. One double-zero-event trial was retained in the descriptive totals but did not contribute to the pooled risk ratio; therefore, 10 trials provided estimable relative effects. The random-effects meta-analysis showed no statistically significant difference between treatment strategies (RR = 1.42; 95% CI: 0.80–2.53; *p* = 0.198). No between-study heterogeneity was observed based on the point estimates (I^2^ = 0%; τ^2^ = 0; Q = 5.64, *p* = 0.775), although the CI for I^2^ was relatively wide (0–62.4%). The CI around the pooled effect included both no difference and potentially clinically relevant effects, precluding a definitive conclusion regarding the relative need for surgery.

#### 3.6.8. All-Cause Mortality

Eleven randomized controlled trials comprising 1634 participants reported all-cause mortality (Figure 10). Death occurred in 21 of 822 participants (2.6%) receiving continuous PPI infusion and in 11 of 812 participants (1.4%) receiving intermittent bolus therapy. Three double-zero-event trials were retained in the descriptive totals but did not contribute to the pooled risk ratio; therefore, eight trials provided estimable relative effects. The random-effects meta-analysis showed no statistically significant difference between treatment strategies (RR = 1.76; 95% CI: 0.90–3.44; *p* = 0.085). No between-study heterogeneity was observed based on the point estimates (I^2^ = 0%; τ^2^ = 0; Q = 4.23, *p* = 0.753), although the CI for I^2^ was wide (0–67.6%). Although the point estimate indicated numerically higher mortality with continuous infusion, the CI included the null effect; therefore, the evidence did not establish a difference between administration strategies.

#### 3.6.9. Patients Meeting the Study-Defined Transfusion Threshold

Five randomized controlled trials comprising 761 participants reported the proportion of patients meeting the study-defined transfusion threshold (Figure 11). The outcome occurred in 190 of 380 participants (50.0%) receiving continuous PPI infusion and in 168 of 381 participants (44.1%) receiving intermittent bolus therapy. The random-effects meta-analysis showed no statistically significant difference between treatment strategies (RR = 1.11; 95% CI: 0.99–1.25; *p* = 0.070). No statistical heterogeneity was observed based on the point estimates (I^2^ = 0%; τ^2^ = 0; Q = 1.41, *p* = 0.842), although the CI for I^2^ was wide (0–79.2%). The point estimate suggested a numerically higher frequency of reaching the transfusion threshold with continuous infusion; however, the CI included the null effect. This exploratory finding should be interpreted cautiously because the operational definition of the transfusion outcome differed across trials.

#### 3.6.10. Blood Units Transfused

Seven randomized controlled trials comprising 1288 participants reported the number of blood units transfused, including 646 participants receiving continuous PPI infusion and 642 receiving intermittent bolus therapy (Figure 12). The random-effects meta-analysis showed no statistically significant difference between treatment strategies (MD = 0.37 units; 95% CI: −0.06 to 0.80; *p* = 0.082). The point estimate suggested a numerically greater transfusion requirement with continuous infusion; however, the CI included the null effect. Moderate between-study heterogeneity was observed based on the point estimate (I^2^ = 47.7%; τ^2^ = 0.102; Q = 11.48, *p* = 0.075), although the CI for I^2^ was wide (0–77.9%).

#### 3.6.11. Length of Hospital Stay

Seven randomized controlled trials comprising 1063 participants reported the length of hospital stay, including 533 participants receiving continuous PPI infusion and 530 receiving intermittent bolus therapy (Figure 13). The random-effects meta-analysis showed no statistically significant difference between treatment strategies (MD = 0.35 days; 95% CI: −0.12 to 0.82; *p* = 0.120). No statistical heterogeneity was observed based on the point estimates (I^2^ = 0%; τ^2^ = 0; Q = 5.75, *p* = 0.451), although the CI for I^2^ was wide (0–70.8%). The point estimate suggested a numerically longer hospital stay with continuous infusion, but the CI included the null effect.

### 3.7. Sensitivity Analyses

All prespecified and additional sensitivity analyses are summarized in Supplementary Table S3. The prespecified time-specific analysis restricted to the five trials reporting rebleeding at 30 days included 792 participants and 56 rebleeding events, with 22 events among 408 participants receiving continuous infusion and 34 among 384 receiving intermittent bolus therapy. The pooled estimate was RR 0.64 (95% CI 0.26–1.57; *p* = 0.241), with low between-study heterogeneity (I^2^ = 20.0%; τ^2^ = 0.057). The confidence interval remained compatible with clinically important effects in either direction. Together with the separate analyses at 24 h, 72 h, and 7 days, this analysis did not materially change the interpretation of the primary study-defined overall-rebleeding synthesis.

In a post-registration sensitivity analysis, excluding Andriulli et al. [20] because of the internal discrepancy in the reported 72-h event counts left four trials comprising 387 participants. The pooled effect remained close to the null, although precision decreased substantially (RR 1.01, 95% CI 0.20–5.00; *p* = 0.991; I^2^ = 35.3%; τ^2^ = 0).

In the post-registration strict post-hemostasis sensitivity analysis excluding Garrido et al. [17], the findings were materially unchanged for overall rebleeding (10 trials, 1580 participants; RR 0.94, 95% CI 0.63–1.41; *p* = 0.746), need for surgery (10 trials, 1591 participants; RR 1.45, 95% CI 0.77–2.74; *p* = 0.216), all-cause mortality (10 trials, 1593 participants; RR 1.71, 95% CI 0.80–3.63; *p* = 0.133), and the study-defined transfusion threshold (4 trials, 720 participants; RR 1.12, 95% CI 0.97–1.30; *p* = 0.089). Garrido et al. [17] did not contribute data to the meta-analyses of mean blood units transfused or length of hospital stay.

The prespecified sensitivity analysis excluding Chiu et al. [18], in which the intermittent-treatment group received scheduled second-look endoscopy and retreatment, did not materially alter the pooled estimate for overall rebleeding (10 trials, 1316 participants; RR 0.93, 95% CI 0.60–1.44; *p* = 0.725). In post-registration extensions of this analysis, exclusion of Chiu et al. [18] likewise did not materially alter the estimates for rebleeding within 24 h (2 trials, 543 participants; RR 0.90, 95% CI 0.001–768.87; *p* = 0.871), rebleeding within 7 days (5 trials, 833 participants; RR 1.08, 95% CI 0.57–2.07; *p* = 0.753), rebleeding at 30 days (4 trials, 487 participants; RR 0.49, 95% CI 0.11–2.17; *p* = 0.226), need for surgery (10 trials, 1327 participants; RR 1.30, 95% CI 0.66–2.58; *p* = 0.400), all-cause mortality (10 trials, 1329 participants; RR 1.48, 95% CI 0.64–3.41; *p* = 0.291), and mean blood units transfused (6 trials, 983 participants; MD 0.39 units, 95% CI −0.18 to 0.95; *p* = 0.138). The 24-h estimate became extremely imprecise because only two trials remained after exclusion. This sensitivity analysis was not applicable to rebleeding within 72 h, the study-defined transfusion threshold, or length of hospital stay because Chiu et al. [18] did not contribute to those quantitative syntheses.

The registered protocol also prespecified sensitivity analyses based on risk-of-bias judgments for the primary outcome. After excluding Hung et al. [16] and Yüksel et al. [21], the two trials judged to have a high overall risk of bias for overall rebleeding, the pooled estimate remained consistent with the primary analysis (9 trials, 1421 participants; RR 0.89, 95% CI 0.57–1.40; *p* = 0.581; I^2^ = 19.3%; τ^2^ = 0.055). The prespecified combined sensitivity analysis that also excluded Chiu et al. [18] because of its asymmetric co-intervention yielded a similar estimate (8 trials, 1116 participants; RR 0.87, 95% CI 0.50–1.52; *p* = 0.581; I^2^ = 28.4%; τ^2^ = 0.103).

The prespecified common-effect Mantel–Haenszel analysis for overall rebleeding yielded a result consistent with the primary random-effects model (11 trials, 1621 participants; RR 0.92, 95% CI 0.68–1.25; *p* = 0.608). Post-registration extensions of this analysis yielded similarly consistent results for rebleeding within 24 h (3 trials, 848 participants; RR 0.84, 95% CI 0.44–1.61; *p* = 0.602), rebleeding within 7 days (6 trials, 1138 participants; RR 1.06, 95% CI 0.74–1.52; *p* = 0.747), and the exploratory 72-h outcome (5 trials, 861 participants; RR 0.92, 95% CI 0.58–1.47; *p* = 0.736). Thus, the interpretation of the findings was unchanged by the choice of statistical model.

Additional post hoc rare-event sensitivity analyses were conducted for the four outcomes whose primary risk-ratio analyses contained at least one zero cell (Table S3). Using common-effect Mantel–Haenszel risk differences calculated without continuity correction, the absolute risk difference was −0.54 percentage points for rebleeding within 72 h (95% CI −4.07 to 2.98; *p* = 0.763), 2.69 percentage points for rebleeding in duodenal ulcers (95% CI −3.97 to 9.36; *p* = 0.428), 0.92 percentage points for surgery (95% CI −0.53 to 2.36; *p* = 0.215), and 1.21 percentage points for all-cause mortality (95% CI −0.12 to 2.55; *p* = 0.075). Trials with zero events in both groups were retained in the surgery and mortality analyses. These findings did not materially alter the interpretation of any outcome.

### 3.8. Assessment of Small-Study Effects

Small-study effects were assessed for overall clinical ulcer rebleeding and need for surgery using contour-enhanced funnel plots and Egger’s linear regression test. For overall clinical ulcer rebleeding (Figure 14a), the funnel plot showed evidence of asymmetry, which was supported by Egger’s regression test (t = −2.51, df = 9, *p* = 0.033; bias estimate = −1.62, SE = 0.64). The negative coefficient indicated that smaller, less precise studies tended to report effect estimates more favorable to continuous PPI infusion. However, this finding should not be interpreted as definitive evidence of publication bias, given the limited number of studies, the low frequency of events, and the possibility that clinical or methodological differences among small trials contributed to the observed asymmetry. For the need for surgery (Figure 14b), Egger’s regression test did not identify statistically significant funnel plot asymmetry (t = −0.14, df = 8, *p* = 0.891; bias estimate = −0.12, SE = 0.81), providing no statistical evidence of small-study effects. Nevertheless, this result should be interpreted cautiously because only 10 studies provided estimable effect sizes and the total number of events was low. Small-study effects were not formally assessed for all-cause mortality because only eight studies provided estimable effect sizes, below the minimum of 10 studies generally recommended for funnel plot asymmetry tests. Formal asymmetry testing was not performed for the 24-h, 72-h, 7-day, or 30-day rebleeding analyses, or for the gastric- and duodenal-ulcer subgroup analyses, because each synthesis included fewer than 10 studies. Accordingly, small-study effects could neither be reliably assessed nor excluded for these outcomes.

### 3.9. Certainty of the Evidence

The certainty of evidence for the seven prespecified critical outcomes was assessed using the GRADE approach (Table 4; Supplementary Table S4). Certainty was rated as very low for overall ulcer rebleeding, rebleeding within 24 h, and rebleeding within 7 days. These ratings were primarily attributable to outcome-specific risk-of-bias concerns and very serious imprecision arising from the limited number of events, failure to meet the optimal information size, and CIs encompassing both clinically important benefit and harm. For overall ulcer rebleeding, the certainty was further reduced because funnel plot asymmetry and Egger’s regression test suggested possible publication bias. Low-certainty evidence was found for the need for surgical intervention and all-cause mortality. Both outcomes were downgraded for risk of bias and imprecision because few events were observed and the CIs included no effect and clinically important harm. Consequently, the very low-certainty evidence for rebleeding and the low-certainty evidence for surgery and all-cause mortality provide limited confidence in the apparent absence of statistically significant differences between treatment strategies, and clinically important effects in either direction cannot be excluded.

The evidence was rated as moderate certainty for the mean number of blood units transfused and length of hospital stay. Both outcomes were downgraded by one level because of risk-of-bias concerns. However, their CIs remained within the prespecified thresholds of clinical importance—±1 blood unit and ±1 hospital day, respectively—supporting the conclusion that continuous infusion probably results in little or no clinically important difference in these outcomes compared with intermittent bolus administration.

No serious concerns regarding indirectness were identified for any outcome, as the populations, interventions, comparators, and outcomes were directly aligned with the review question. Similarly, inconsistency was not considered serious because statistical heterogeneity was absent or moderate and did not produce materially conflicting clinical interpretations.

## 4. Discussion

This systematic review and meta-analysis included 12 unique randomized controlled trials reported in 13 publications. Across overall ulcer rebleeding, rebleeding within 24 h or 7 days, need for surgery, all-cause mortality, transfusion requirements, and length of hospital stay, the pooled analyses did not demonstrate statistically significant superiority of continuous intravenous PPI infusion over intermittent intravenous bolus therapy. This absence of statistically significant differences should not be interpreted as evidence of equivalence or noninferiority. The certainty of evidence was very low for the rebleeding outcomes and low for surgery and mortality, largely because of outcome-specific risk-of-bias concerns, sparse events, and CIs that did not exclude clinically important benefit or harm. In contrast, moderate-certainty evidence indicated that continuous infusion probably results in little or no clinically important difference in the number of blood units transfused or length of hospital stay.

The rationale for continuous PPI infusion is based on the relationship between intragastric acidity and clot stability. Experimental evidence indicates that acid and pepsin impair platelet aggregation, blood coagulation, and clot integrity, providing a biological basis for maintaining sustained gastric acid suppression after successful endoscopic hemostasis [3]. Continuous infusion was therefore expected to provide more stable inhibition of newly activated proton pumps and maintain intragastric pH within a range favorable to clot formation. However, this theoretical pharmacodynamic advantage did not translate into a measurable clinical benefit in the present analysis. One possible explanation is that sufficiently frequent intermittent intravenous administration provides adequate acid suppression during the clinically critical period. Moreover, the intragastric pH required to prevent clinically relevant clot dissolution may be lower than the historically proposed target of approximately 6, although the available trials were not designed to establish an optimal pharmacodynamic threshold [3,7].

The findings are broadly consistent with the systematic review and noninferiority meta-analysis by Sachar et al., which concluded that intermittent PPI therapy was not inferior to the conventional bolus-plus-continuous-infusion regimen for recurrent bleeding after endoscopic treatment of high-risk ulcers [7]. However, several methodological differences should be considered. Sachar et al. evaluated a broader range of intermittent regimens, including oral and intravenous administration, and used a predefined noninferiority margin based primarily on absolute risk differences. In contrast, the present review was restricted to direct comparisons between continuous and intermittent intravenous administration, included only full-text randomized trials, incorporated evidence published through 2024, and applied random-effects models with Hartung–Knapp adjustment. Furthermore, risk of bias was evaluated separately for each outcome using RoB 2, and the certainty of evidence was formally assessed using GRADE. Consequently, although both reviews reach a similar overall clinical interpretation, the present analysis emphasizes that the available intravenous evidence remains insufficiently precise to establish definitive equivalence across all patient-important outcomes.

Current clinical guidelines reflect this evolving evidence base. The American College of Gastroenterology recommends high-dose PPI therapy administered either continuously or intermittently for three days after successful endoscopic hemostasis [5]. The European Society of Gastrointestinal Endoscopy continues to identify an 80-mg intravenous bolus followed by an 8-mg/h infusion for 72 h as a standard regimen but recognizes high-dose intermittent intravenous or oral therapy as an acceptable alternative [6]. In contrast, the International Consensus Group more explicitly recommends an intravenous loading dose followed by continuous infusion for patients with high-risk stigmata after successful endoscopic treatment [29]. This variation illustrates that continuous infusion remains an established reference strategy, while intermittent treatment has progressively gained acceptance. The present findings support the availability of intermittent intravenous therapy as a reasonable clinical option but do not establish that every intermittent dose or frequency is interchangeable with the standard continuous regimen.

The estimates for surgery and mortality require particularly cautious interpretation. Although neither outcome differed statistically between treatment groups, the pooled point estimates were numerically higher with continuous infusion: RR 1.42 for surgery and RR 1.76 for mortality. These results do not demonstrate that continuous infusion increases the risk of either outcome because both CIs included the null effect. However, the intervals also encompassed clinically important harm, and the total number of events was small. Accordingly, the correct interpretation is not that the two strategies have identical effects, but that the available evidence failed to establish a difference and remains insufficient to exclude potentially important effects. This distinction is reflected in the low GRADE certainty assigned to both outcomes.

The evidence was more informative for blood utilization and hospitalization. The pooled mean difference was 0.37 additional blood units with continuous infusion, with a 95% CI ranging from 0.06 fewer to 0.80 more units. Similarly, length of stay was 0.35 days longer, with a CI ranging from 0.12 days shorter to 0.82 days longer. Both intervals remained within the prespecified thresholds of ±1 blood unit and ±1 hospital day. Therefore, moderate-certainty evidence supports the conclusion that continuous infusion probably produces little or no clinically important difference in these outcomes. The exploratory analysis of the proportion of patients meeting study-defined transfusion thresholds should be interpreted less confidently because transfusion criteria varied across trials and may have been influenced by local practices, baseline hemoglobin values, comorbidities, and clinician judgment.

Clinical and methodological diversity among the included trials also affects interpretation. Although 10 trials used the conventional continuous regimen of an 80-mg loading dose followed by 8 mg/h for 72 h, intermittent schedules ranged from 40 mg once daily to administration every 6, 8, or 12 h [16,17,18,19,20,21,22,23,24,25,26,27]. Pantoprazole was used in most studies, whereas two trials used omeprazole and one allowed either agent. Follow-up periods ranged from 72 h to 30 days, and definitions of recurrent bleeding were not completely uniform. Accordingly, the primary study-defined overall-rebleeding synthesis should be interpreted as a pooled result across each trial’s longest reported clinical follow-up rather than as an effect estimate at a single standardized time point. The additional analysis restricted to trials reporting 30-day rebleeding did not materially change the interpretation, although its confidence interval remained wide. Endoscopic management also evolved across trials and included injection, thermal, and mechanical techniques in different combinations. Of particular relevance, Chiu et al. incorporated scheduled second-look endoscopy and retreatment exclusively into the intermittent-treatment strategy, meaning that its comparator represented a combined pharmacological and procedural intervention [18]. Consequently, this review cannot identify the optimal intermittent dose or dosing frequency, nor determine whether treatment effects are consistent across specific endoscopic risk categories.

Statistical heterogeneity was low or absent for most outcomes, but this should not be equated with complete clinical homogeneity. When the number of studies and events is limited, heterogeneity tests have low power, and wide CIs around I^2^ estimates may remain compatible with meaningful between-study variability. Conversely, the moderate heterogeneity observed for the number of blood units transfused did not lead to materially conflicting clinical interpretations because the individual and pooled effects generally remained close to the threshold of no clinically important difference. Thus, the principal limitations of the evidence were not substantial statistical inconsistency, but methodological uncertainty, sparse outcome events, and insufficient information size.

The outcome-specific RoB 2 assessments showed that most trial results raised some concerns, while relatively few were judged to be at low risk of bias. Limitations frequently involved incomplete reporting of allocation procedures, absence of accessible prespecified analysis plans, and uncertainty regarding the selection of reported results. These concerns were particularly important for subjective or variably defined outcomes such as clinical rebleeding. Funnel plot asymmetry and Egger’s regression suggested possible small-study effects for overall rebleeding, with smaller studies tending to report estimates more favorable to continuous infusion. However, this finding does not establish publication bias because, with only 11 studies and sparse events, asymmetry testing has limited ability to distinguish publication bias from chance, clinical heterogeneity, or methodological differences. Conversely, the absence of statistically significant asymmetry for surgery cannot reliably exclude small-study effects because only 10 studies provided estimable effect sizes and the total number of events was low. Formal asymmetry testing was not appropriate for the 24-h (three trials), 72-h (five trials), 7-day (six trials), and 30-day (five trials) rebleeding analyses or for the gastric- and duodenal-ulcer subgroup analyses (three trials each). These syntheses also produced wide CIs compatible with clinically important benefit and harm. Consequently, possible small-study effects reduce confidence in the primary overall-rebleeding estimate, whereas the limited number of studies and substantial imprecision render the time-specific and ulcer-location findings inconclusive rather than evidence of no difference.

From a clinical perspective, the results do not provide a compelling basis for routinely preferring continuous infusion solely to improve rebleeding, surgery, mortality, transfusion, or hospital-stay outcomes. Intermittent intravenous administration may therefore be a reasonable alternative after successful endoscopic hemostasis, particularly where infusion pumps, intravenous access, pharmacy preparation, or continuous nursing supervision are limited. Observational economic evidence suggests that intermittent strategies may reduce treatment costs and resource utilization, although these findings cannot be directly attributed to the randomized evidence synthesized in this review [30]. Because formal cost-effectiveness, nursing workload, patient mobility, and treatment acceptability were not evaluated in the included trials, these potential advantages should be regarded as implementation considerations rather than proven findings of the present meta-analysis. Treatment selection should also consider the endoscopic risk profile, comorbidities, institutional protocols, and the dose and frequency of the proposed intermittent regimen.

This review has several strengths. It used a comprehensive search across five electronic sources without year or language restrictions, included only randomized controlled trials directly comparing eligible intravenous strategies, and distinguished 12 unique trials from 13 linked reports to avoid double counting participants. Rebleeding was analyzed according to clinically relevant time windows, while exploratory and ulcer-location analyses were separated from the prespecified critical outcomes. Random-effects models with restricted maximum-likelihood estimation and Hartung–Knapp adjustment were used, and double-zero-event trials were retained in descriptive totals without forcing them into relative-effect calculations. The review also integrated outcome-specific RoB 2 assessments, small-study-effect analyses when sufficiently supported, GRADE evaluation, and explicit thresholds of clinical importance for continuous outcomes.

Several limitations should nevertheless be acknowledged. Many trials were small, and the low frequency of rebleeding, surgery, and death resulted in wide CIs and failure to meet the optimal information size for several outcomes. The trials varied in intermittent dosing, PPI selection, loading-dose administration, endoscopic treatment, co-interventions, outcome definitions, and follow-up duration. In Garrido et al. [17], randomization occurred before endoscopy, and endoscopic hemostatic therapy was not administered to all randomized participants; however, exclusion of this trial in sensitivity analyses did not materially alter the pooled estimates for any outcome to which it contributed. Most were single-center studies, and several were conducted before contemporary endoscopic rescue modalities became widely available. Although restricting eligibility to full-text articles allowed complete assessment of study methods and outcomes, excluding conference abstracts and other reports without a full-text publication may have introduced reporting bias by omitting potentially relevant data that were not published in full. Patient-level data were unavailable, preventing reliable evaluation of treatment effects according to Forrest classification, ulcer size and location, hemodynamic instability, comorbidity burden, or validated rebleeding-risk scores. The review was also not designed as a formal noninferiority analysis; therefore, failure to detect statistically significant superiority cannot establish equivalence or noninferiority. Finally, possible small-study effects for overall rebleeding further reduce confidence in the primary efficacy finding.

Future trials should use adequately powered multicenter noninferiority designs, clinically justified margins, concealed randomization, blinded outcome adjudication, and prospectively registered protocols. Rebleeding definitions and assessment windows should be standardized, with early rebleeding, 7-day rebleeding, 30-day mortality, surgery, transcatheter arterial embolization, transfusion, and length of stay reported consistently [31]. Such trials should directly compare clearly defined high-dose intermittent intravenous regimens with the standard bolus-plus-infusion regimen and stratify randomization according to clinically relevant endoscopic risk. They should also incorporate formal economic evaluations using context-specific medication-acquisition, staffing, infrastructure, and healthcare-utilization costs, together with infusion-pump requirements, nursing workload, intravenous-line use, patient mobility, and treatment acceptability, to determine whether noninferiority can be established and, if so, whether either administration strategy offers operational or economic advantages across different healthcare systems.

## 5. Conclusions

Continuous intravenous PPI infusion was not shown to be superior to intermittent intravenous bolus therapy following successful endoscopic hemostasis for bleeding peptic ulcers. Moderate-certainty evidence indicates that continuous infusion probably results in little or no clinically important difference in the number of blood units transfused or length of hospital stay. However, for rebleeding, surgery, and all-cause mortality, the very low- to low-certainty evidence establishes neither equivalence nor noninferiority and does not exclude clinically important benefit or harm. Because the intermittent regimens differed substantially across the included trials, this review cannot determine the optimal intermittent dose or dosing frequency. Adequately powered multicenter noninferiority trials with standardized outcome definitions are needed to clarify comparative effects on infrequent serious outcomes and to directly evaluate resource use and cost-effectiveness.

## Figures and Tables

**Figure 1:**
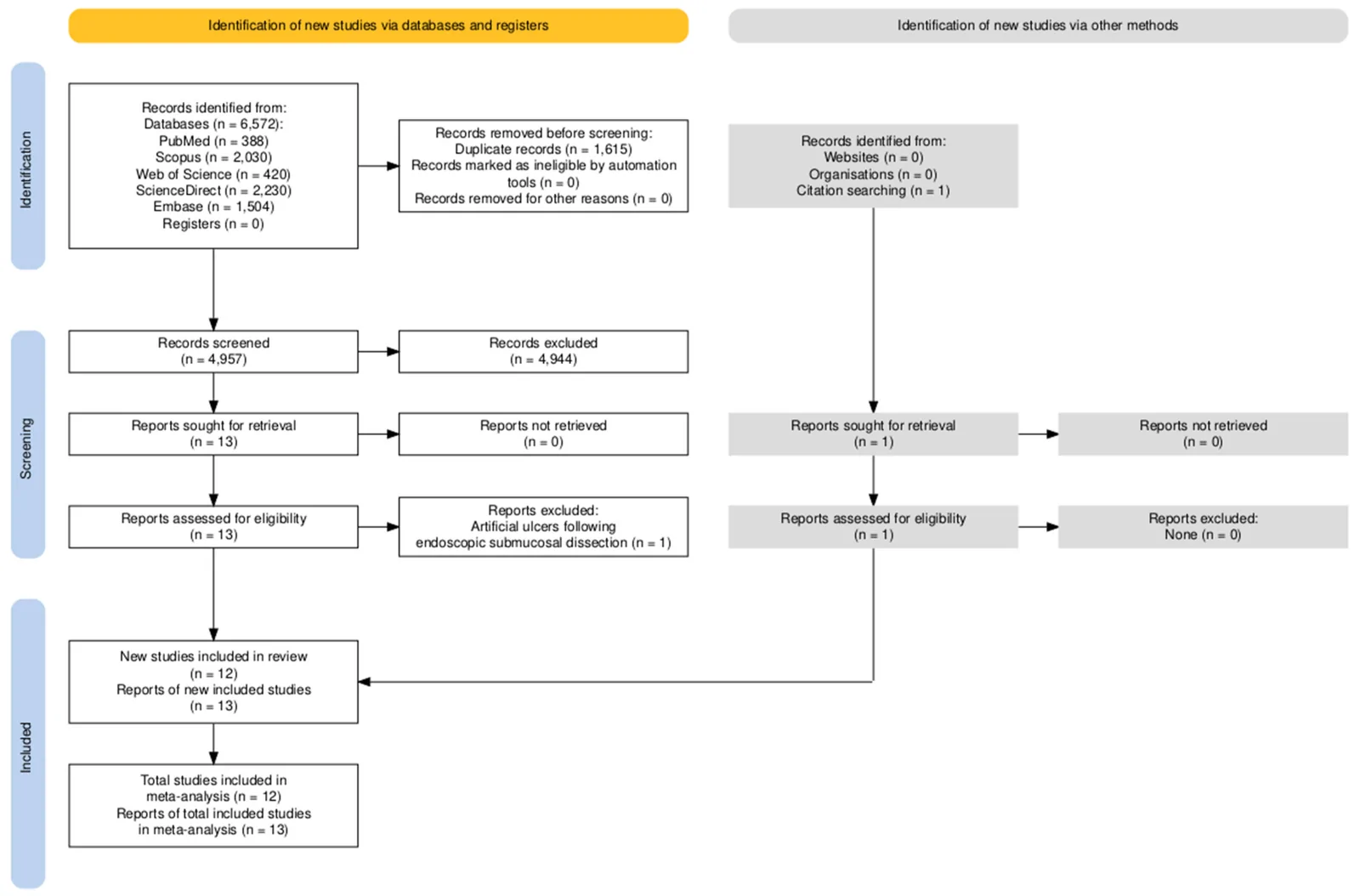
PRISMA 2020 flow diagram illustrating the study identification, screening, eligibility assessment, and inclusion process for randomized controlled trials comparing continuous intravenous PPI infusion with intermittent intravenous bolus therapy after endoscopic hemostasis for bleeding peptic ulcers.

**Figure 2:**
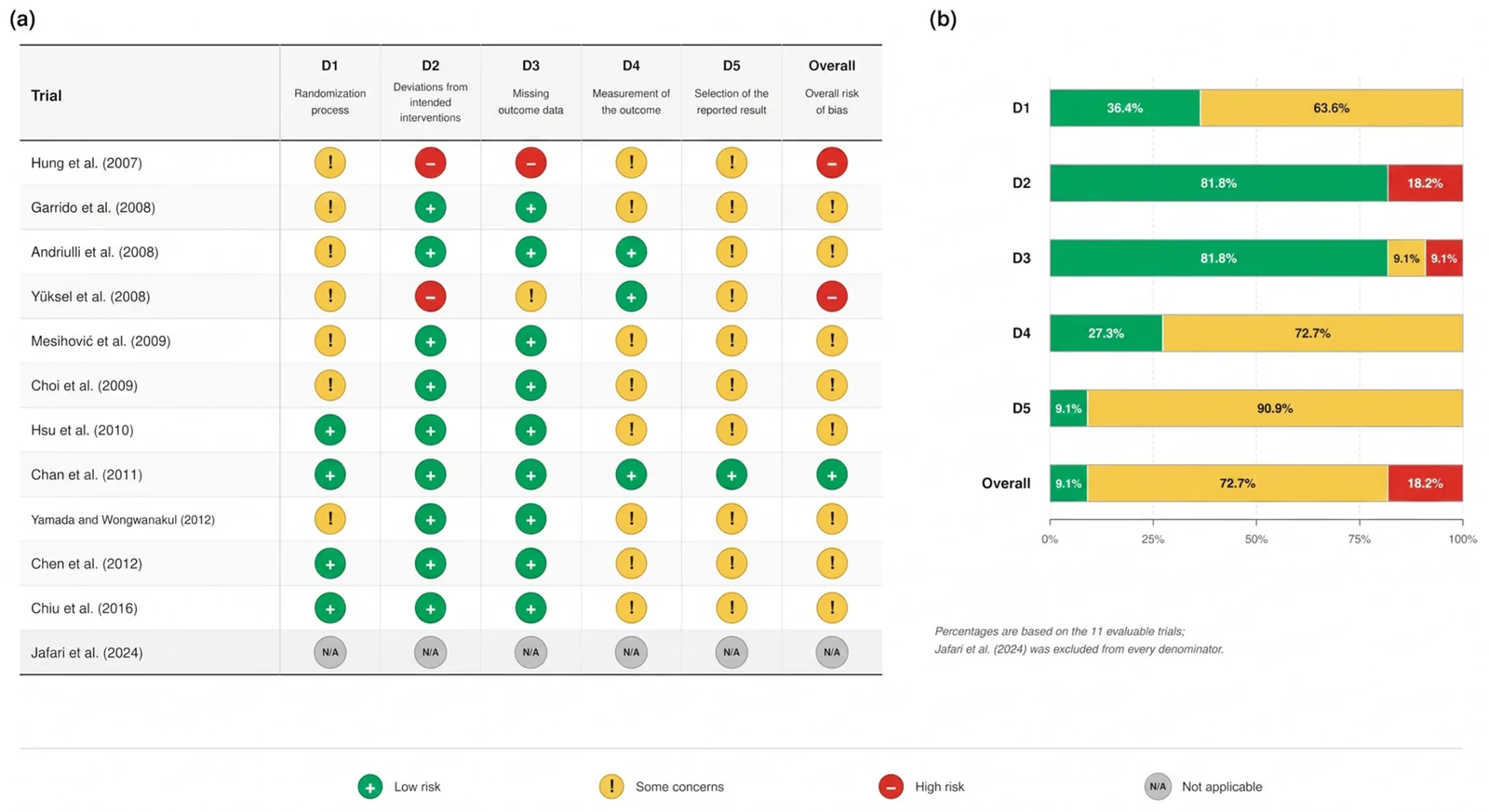
Risk-of-bias assessment for the primary outcome of overall ulcer rebleeding at the longest follow-up reported by each trial. (**a**) Trial-level judgments across the five domains of the Cochrane Risk of Bias 2 tool and the overall assessment. Jafari et al. [19] was classified as not applicable because the study did not report a clinically defined rebleeding outcome compatible with the primary outcome. (**b**) Percentage distribution of risk-of-bias judgments across the 11 evaluable trials [16,17,18,20,21,22,23,24,25,26,27]. Green indicates low risk, yellow indicates some concerns, red indicates high risk, and gray indicates not applicable. D1, bias arising from the randomization process; D2, bias due to deviations from intended interventions; D3, bias due to missing outcome data; D4, bias in measurement of the outcome; D5, bias in selection of the reported result. Created in BioRender. Carrillo, J. (2026) https://BioRender.com/i6mvli4.

**Figure 3:**
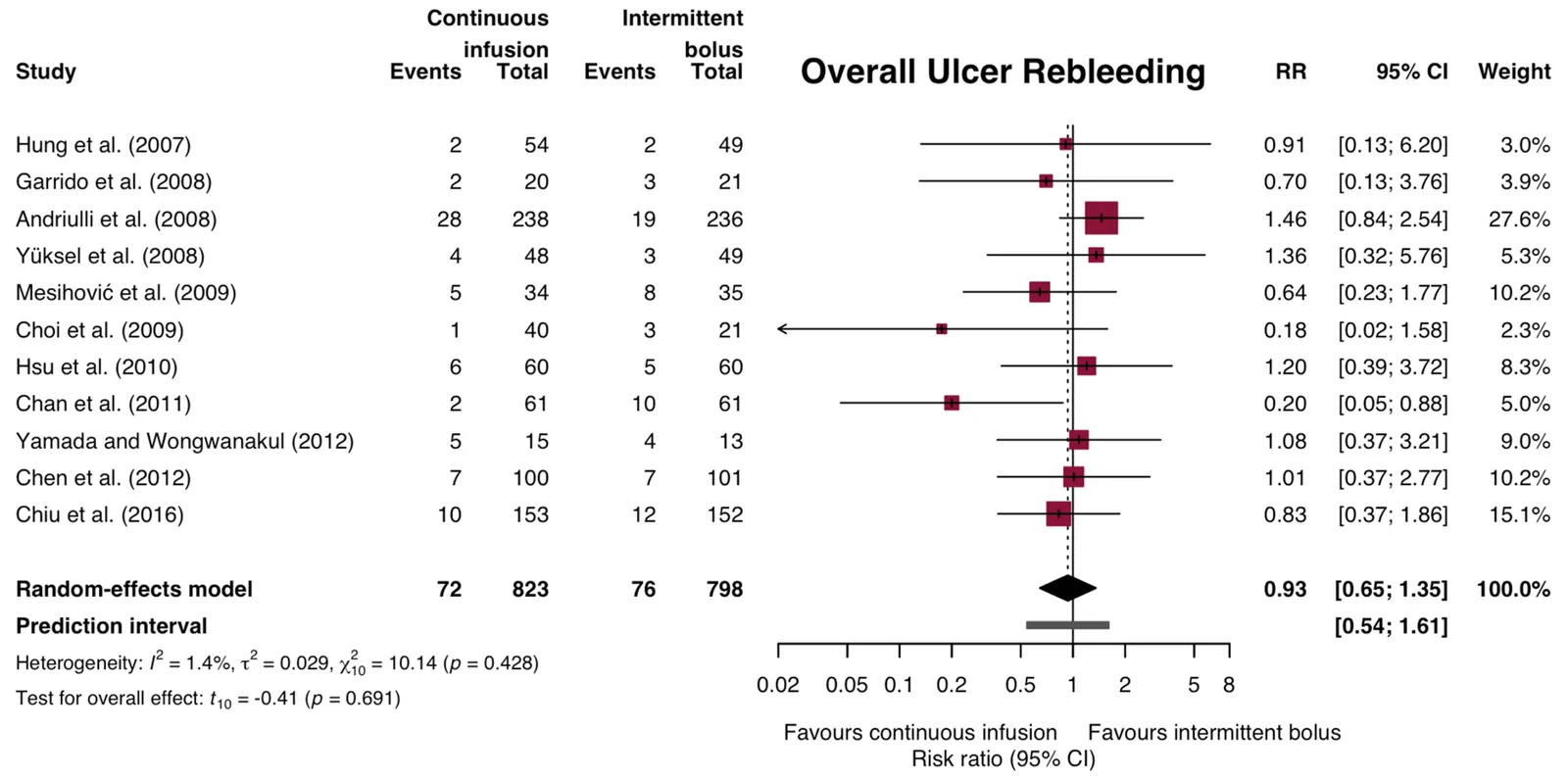
Forest plot comparing continuous proton pump inhibitor infusion with intermittent bolus therapy for overall ulcer rebleeding at the longest follow-up reported by each trial [16,17,18,20,21,22,23,24,25,26,27]. Risk ratios and 95% CIs were pooled using a random-effects model with restricted maximum-likelihood estimation and the Hartung–Knapp adjustment. The horizontal line beneath the pooled estimate represents the 95% prediction interval. RR < 1 favors continuous infusion, whereas RR > 1 favors intermittent bolus therapy.

**Figure 4:**
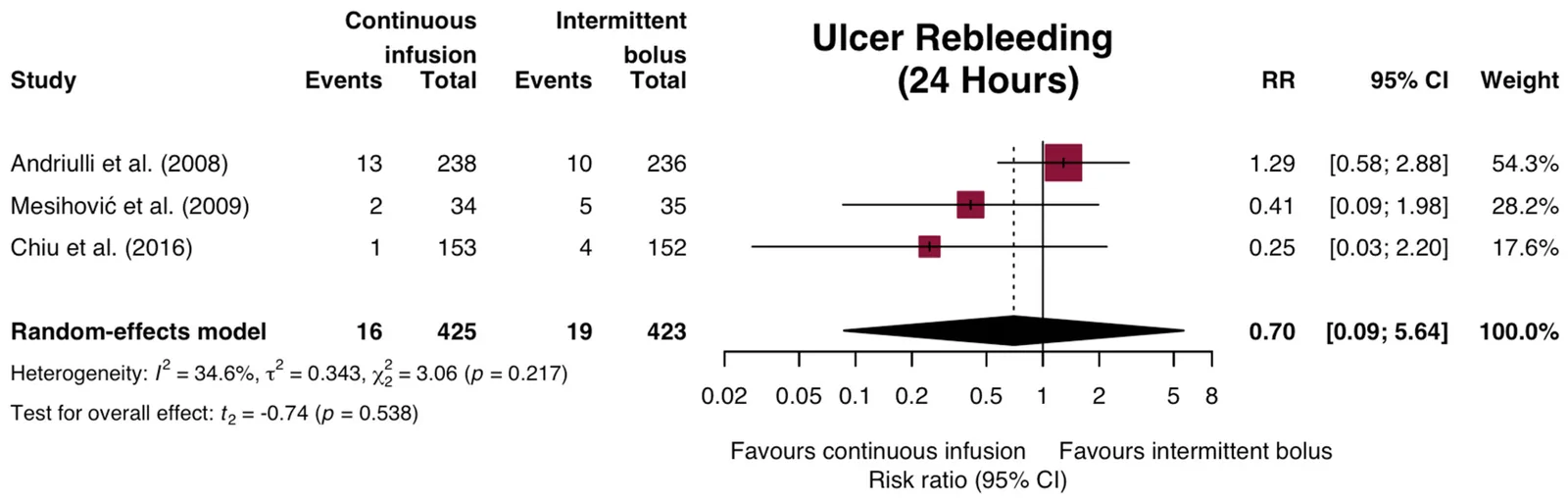
Forest plot comparing continuous proton pump inhibitor infusion with intermittent bolus therapy for clinical ulcer rebleeding within 24 h [18,20,22]. Risk ratios and 95% CIs were pooled using a random-effects model with restricted maximum-likelihood estimation and the Hartung–Knapp adjustment. RR < 1 favors continuous infusion, whereas RR > 1 favors intermittent bolus therapy.

**Figure 5:**
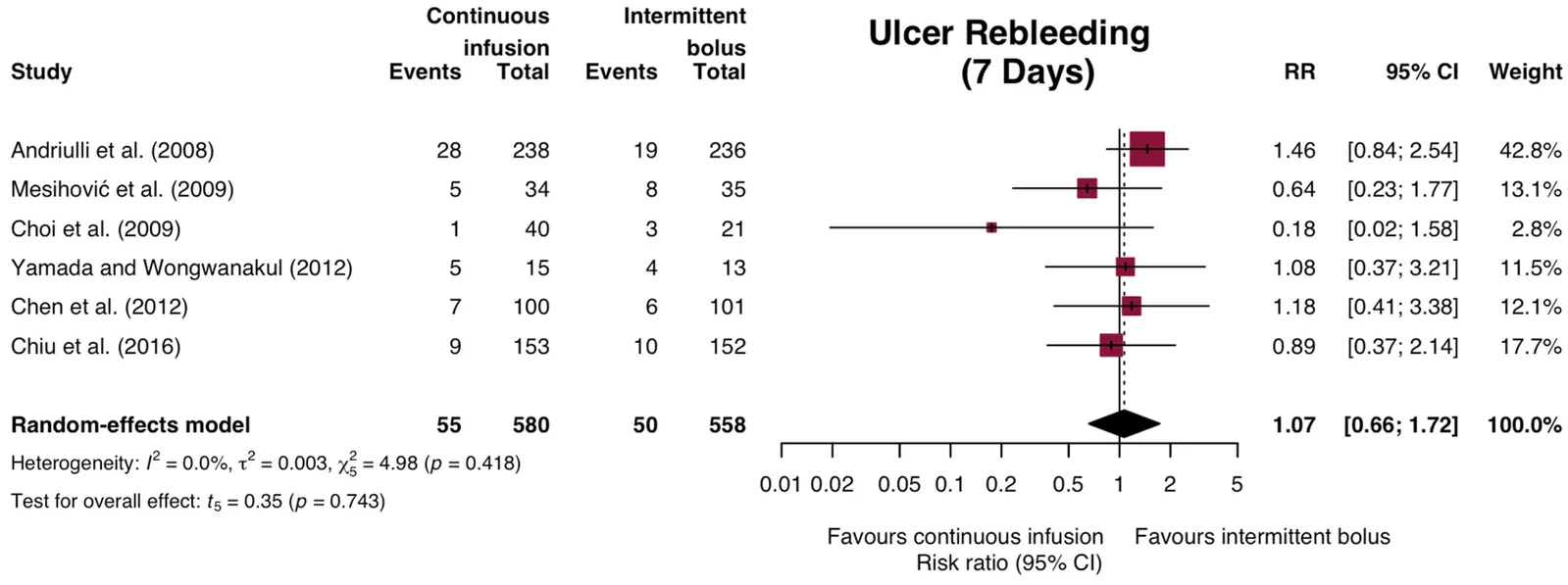
Forest plot comparing continuous proton pump inhibitor infusion with intermittent bolus therapy for clinical ulcer rebleeding within seven days [18,20,22,23,26,27]. Risk ratios and 95% CIs were pooled using a random-effects model with restricted maximum-likelihood estimation and the Hartung–Knapp adjustment. RR < 1 favors continuous infusion, whereas RR > 1 favors intermittent bolus therapy.

**Figure 6:**
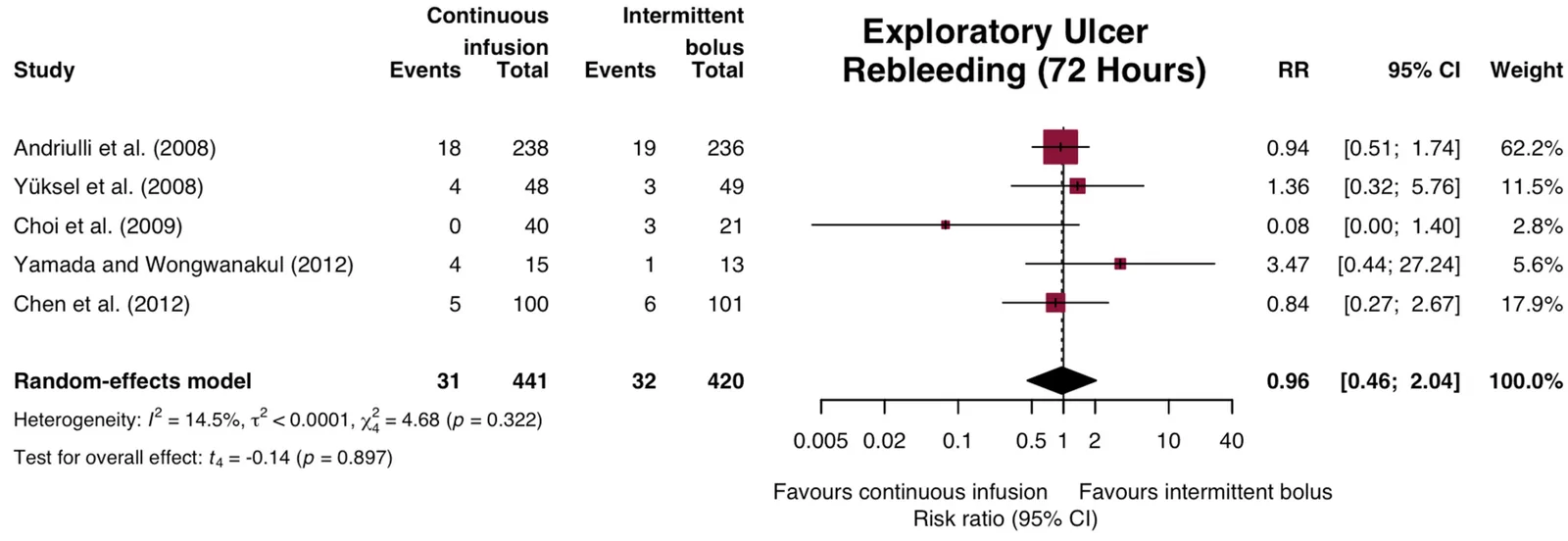
Forest plot comparing continuous proton pump inhibitor infusion with intermittent bolus therapy for clinical ulcer rebleeding within 72 h [20,21,23,26,27]. This exploratory analysis included event counts reported within 72 h or the first three days, as well as events that could be unambiguously classified within this time window from their reported timing. Risk ratios and 95% CIs were pooled using a random-effects model with restricted maximum-likelihood estimation and the Hartung–Knapp adjustment. A continuity correction was applied to the study containing a zero cell frequency. RR < 1 favors continuous infusion, whereas RR > 1 favors intermittent bolus therapy.

**Figure 7:**
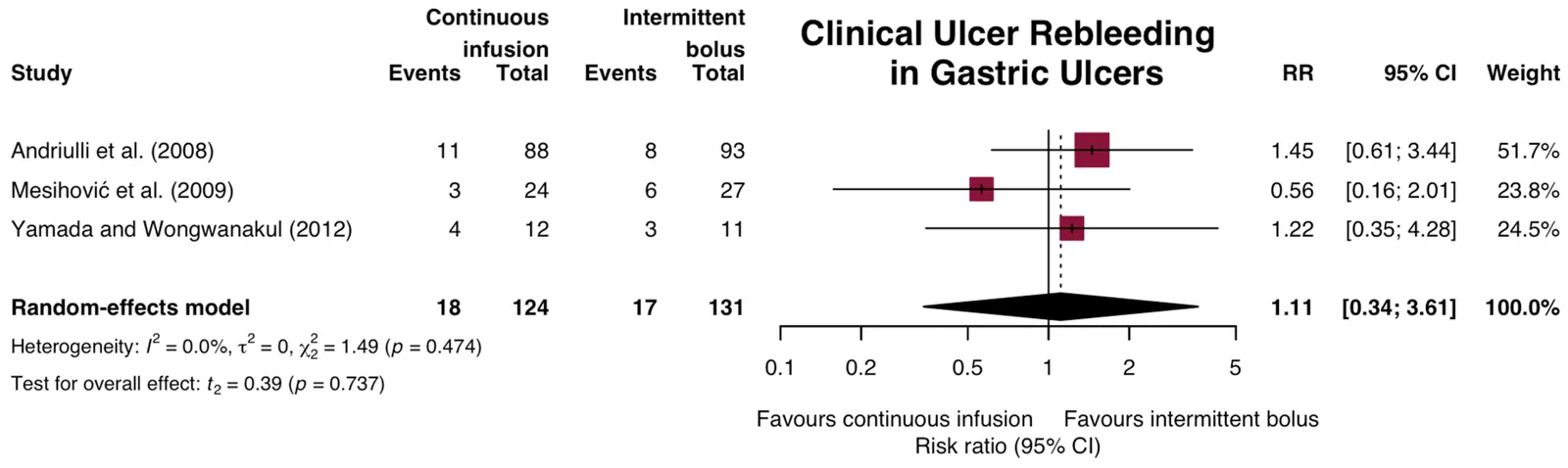
Forest plot comparing continuous proton pump inhibitor infusion with intermittent bolus therapy for clinical rebleeding in gastric ulcers [20,22,26]. Risk ratios and 95% CIs were pooled using a random-effects model with restricted maximum-likelihood estimation and the Hartung–Knapp adjustment. RR < 1 favors continuous infusion, whereas RR > 1 favors intermittent bolus therapy.

**Figure 8:**
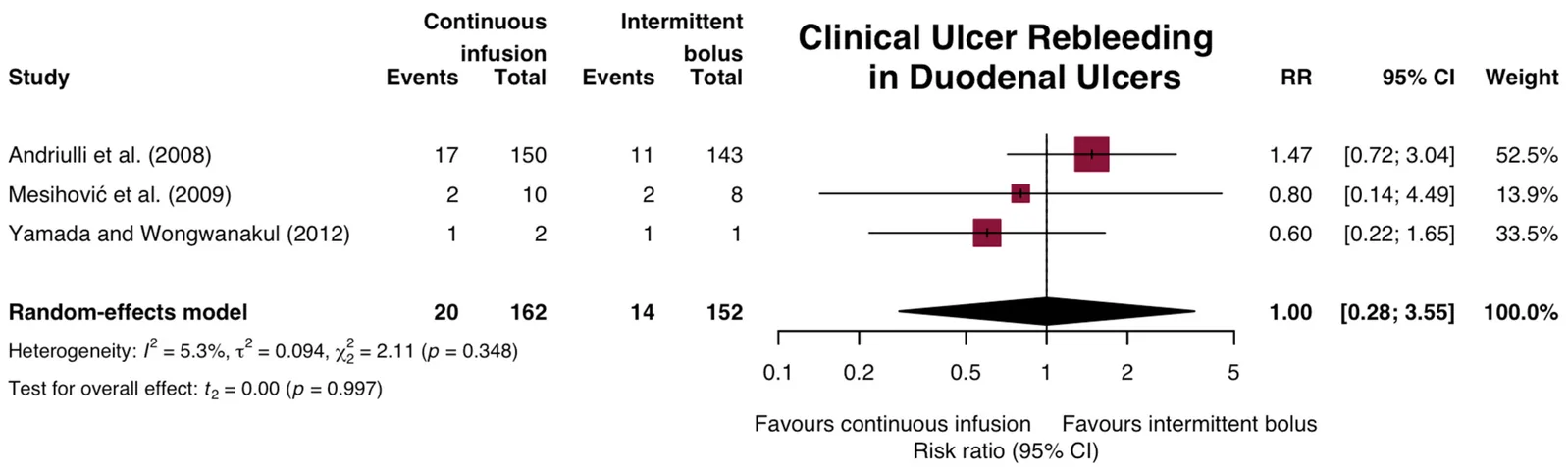
Forest plot comparing continuous proton pump inhibitor infusion with intermittent bolus therapy for clinical rebleeding in duodenal ulcers [20,22,26]. This exploratory subgroup analysis included three randomized controlled trials. Risk ratios and 95% CIs were pooled using a random-effects model with restricted maximum-likelihood estimation and the Hartung–Knapp adjustment. A continuity correction was applied to the study containing a zero cell frequency. RR < 1 favors continuous infusion, whereas RR > 1 favors intermittent bolus therapy.

**Figure 9:**
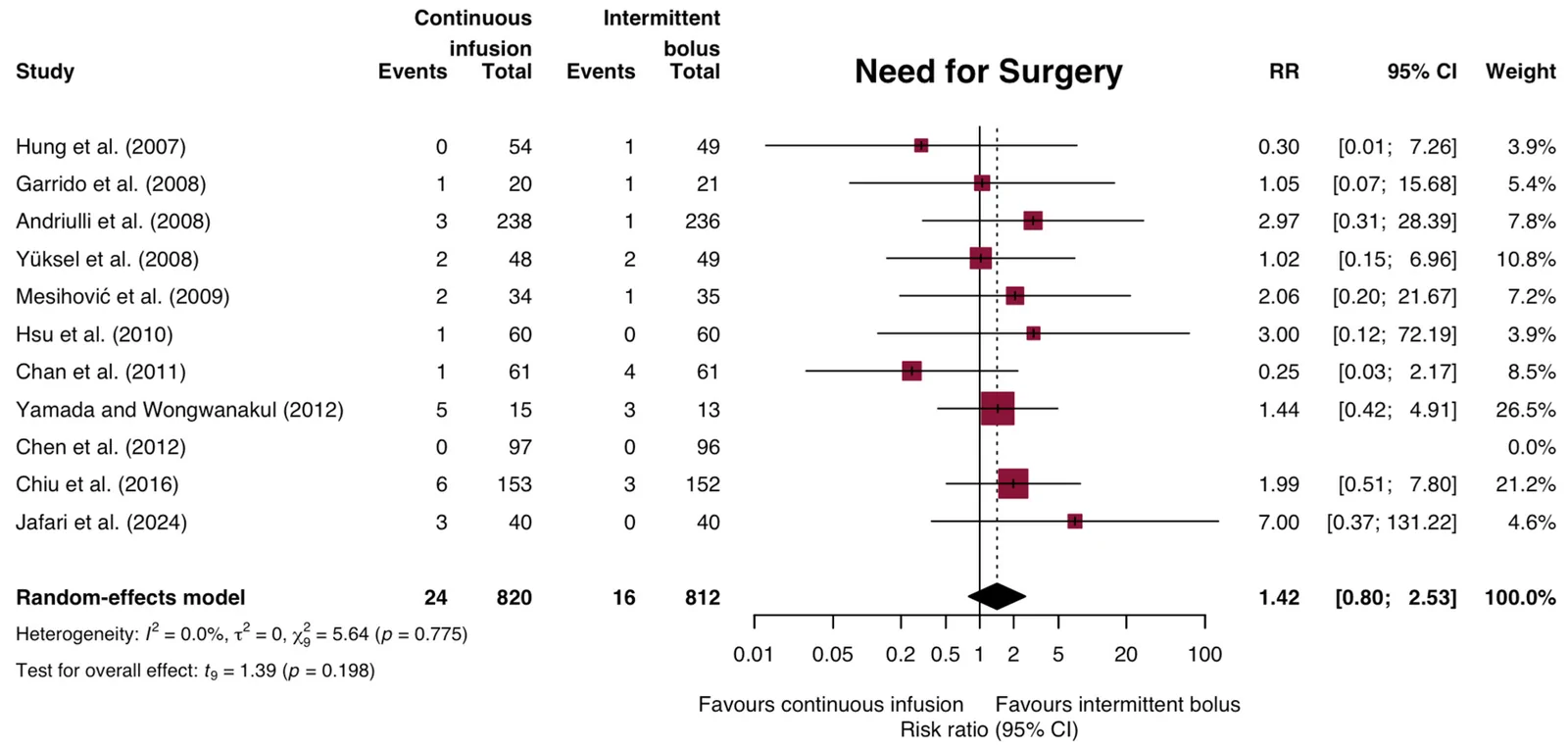
Forest plot comparing continuous proton pump inhibitor infusion with intermittent bolus therapy for the need for surgery [16,17,18,19,20,21,22,24,25,26,27]. Risk ratios and 95% CIs were pooled using a random-effects model with restricted maximum-likelihood estimation and the Hartung–Knapp adjustment. Continuity corrections were applied to studies with zero cell frequencies. Chen et al. [27], which reported zero events in both treatment groups, was retained in the descriptive totals but did not contribute to the pooled effect estimate. RR < 1 favors continuous infusion, whereas RR > 1 favors intermittent bolus therapy.

**Figure 10:**
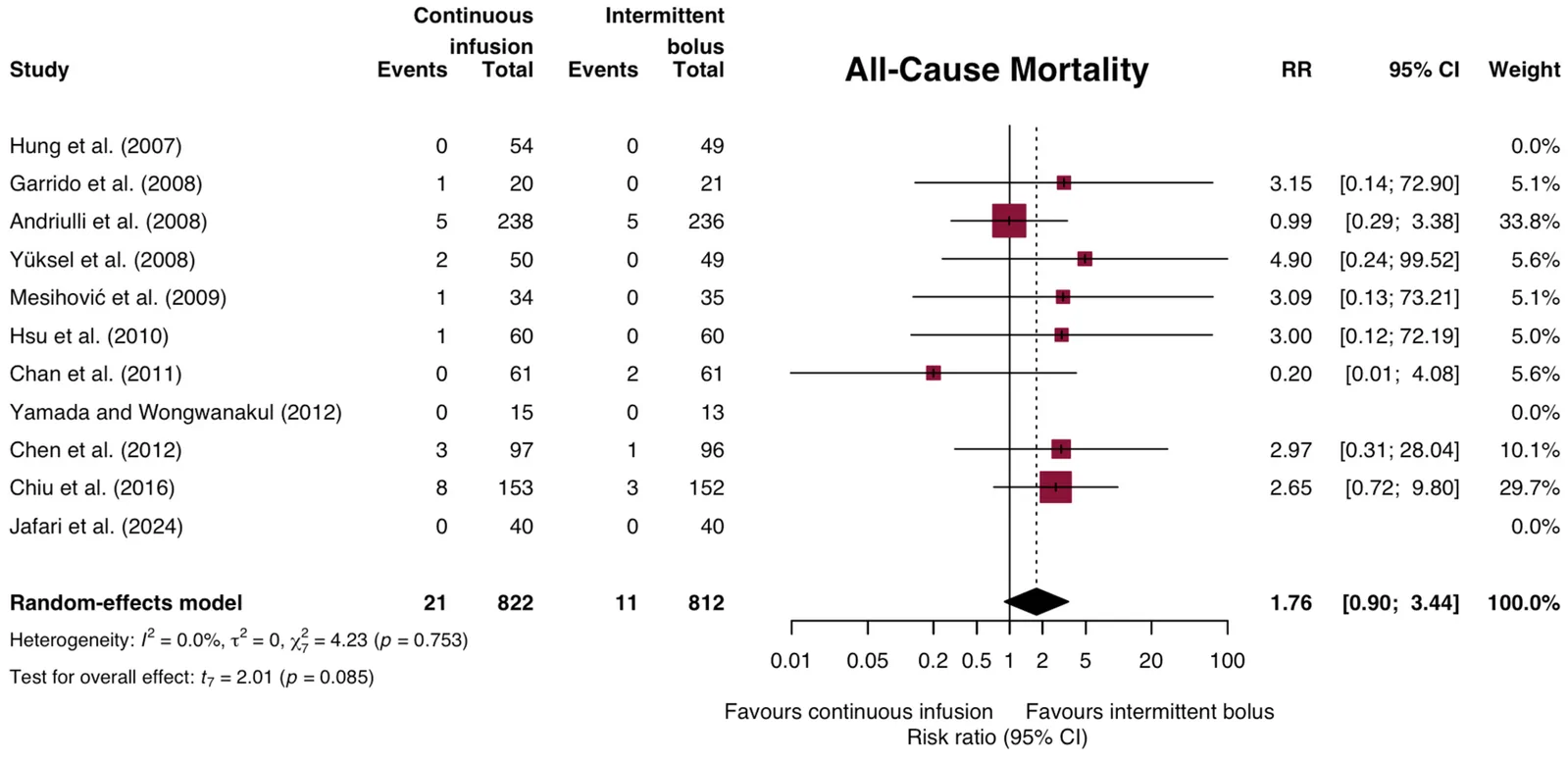
Forest plot comparing continuous proton pump inhibitor infusion with intermittent bolus therapy for all-cause mortality [16,17,18,19,20,21,22,24,25,26,27]. Risk ratios and 95% CIs were pooled using a random-effects model with restricted maximum-likelihood estimation and the Hartung–Knapp adjustment. Continuity corrections were applied to studies with zero cell frequencies. Three trials reporting zero deaths in both treatment groups were retained in the descriptive totals but did not contribute to the pooled effect estimate. RR < 1 favors continuous infusion, whereas RR > 1 favors intermittent bolus therapy.

**Figure 11:**
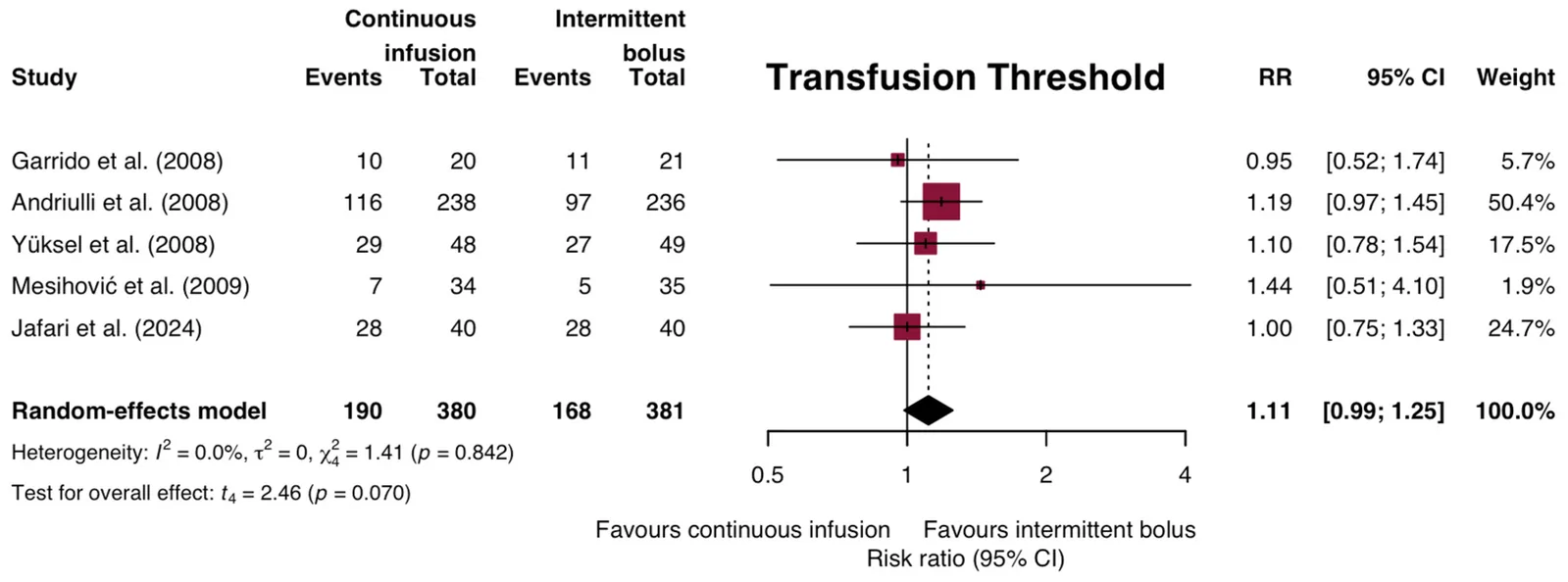
Forest plot comparing continuous proton pump inhibitor infusion with intermittent bolus therapy for the proportion of patients meeting the study-defined transfusion threshold [17,19,20,21,22]. Risk ratios and 95% CIs were pooled using a random-effects model with restricted maximum-likelihood estimation and the Hartung–Knapp adjustment. The operational definition of the transfusion outcome varied across trials; therefore, the pooled result should be considered exploratory. RR < 1 favors continuous infusion, whereas RR > 1 favors intermittent bolus therapy.

**Figure 12:**
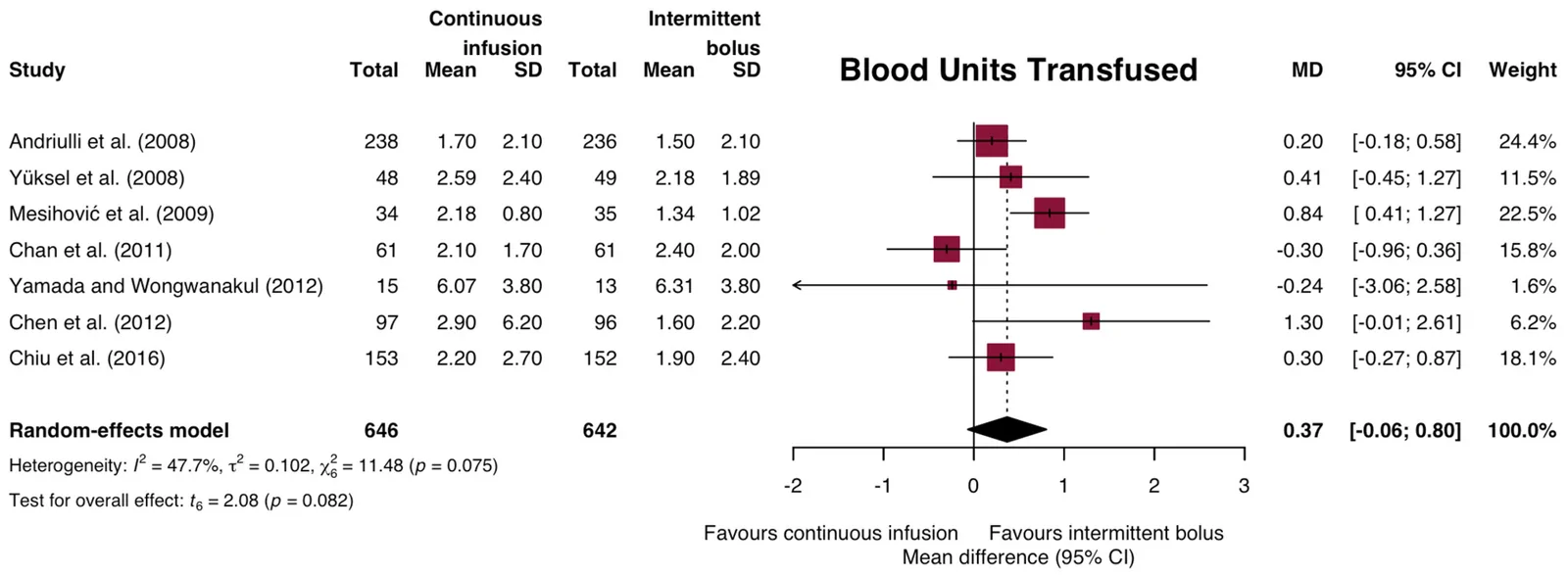
Forest plot comparing continuous proton pump inhibitor infusion with intermittent bolus therapy for the number of blood units transfused [18,20,21,22,25,26,27]. Mean differences (MDs) and 95% CIs were pooled using a random-effects model with restricted maximum-likelihood estimation and the Hartung–Knapp adjustment. Negative MD values favor continuous infusion, whereas positive MD values favor intermittent bolus therapy.

**Figure 13:**
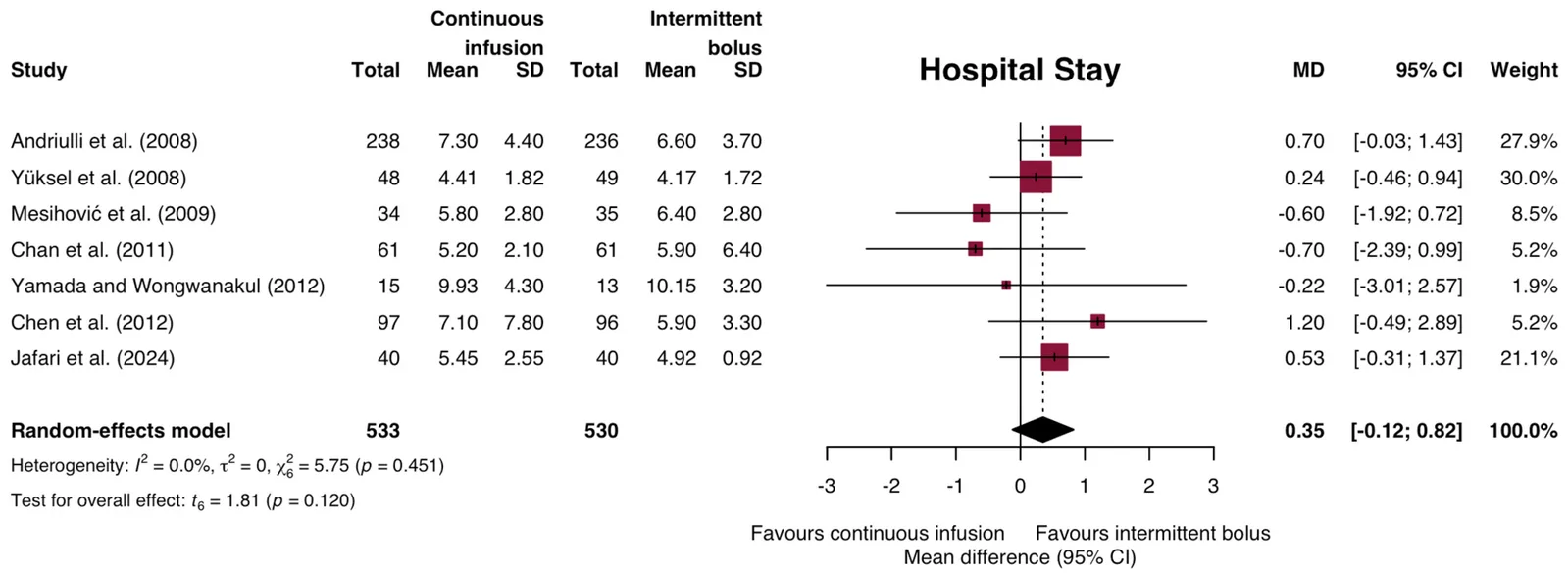
Forest plot comparing continuous proton pump inhibitor infusion with intermittent bolus therapy for length of hospital stay [19,20,21,22,25,26,27]. Mean differences (MDs) and 95% CIs were pooled using a random-effects model with restricted maximum-likelihood estimation and the Hartung–Knapp adjustment. Negative MD values favor continuous infusion, whereas positive MD values favor intermittent bolus therapy.

**Figure 14:**
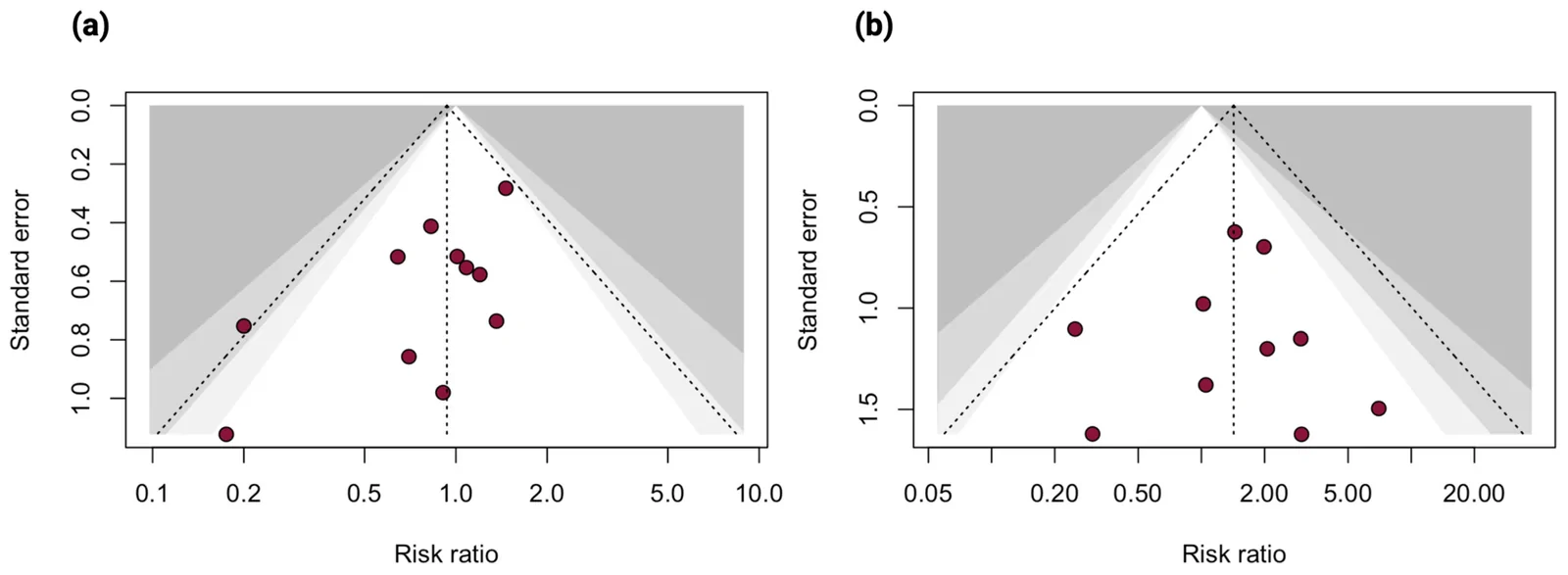
Contour-enhanced funnel plots for the assessment of small-study effects. Funnel plots are presented for (**a**) overall clinical ulcer rebleeding and (**b**) need for surgery. Each point represents an individual study plotted according to its risk ratio and standard error. The vertical line represents the pooled random-effects estimate, and the shaded regions indicate different levels of statistical significance. Egger’s regression test indicated statistically significant asymmetry for overall clinical ulcer rebleeding (*p* = 0.033), suggesting potential small-study effects, whereas no statistically significant asymmetry was detected for the need for surgery (*p* = 0.891). These findings should be interpreted cautiously because of the limited number of informative studies and the low event frequency. Created in BioRender. Carrillo, J. (2026) https://BioRender.com/3f55zjw.

**Table 1:** (**A**) General characteristics of the included randomized controlled trials. (**B**) Clinical and treatment characteristics of the included randomized controlled trials.

(**A**)						
**Study**	**Country/** **Region**	**Study Design**	**Registration**	**Study Population**	**Sample Size, Total** **(Continuous/** **Bolus)**	**Mean Age, Years** **(Continuous/** **Bolus)**
Hung et al. (2007) [16]	Hong Kong, China	Randomized, single-center, three-arm trial	NR	Patients with bleeding peptic ulcers and major stigmata after successful endoscopic hemostasis; only the continuous-infusion and bolus arms were eligible	114 randomized (58/56); 103 analyzed (54/49)	63.7/57.8; SD NR
Garrido et al. (2008) [17]	Spain	Prospective, randomized, single-center trial	NR	Consecutive patients with high-risk ulcer-related upper gastrointestinal bleeding	41 (20/21)	60.1 ± 16.8/60.7 ± 13.3
Andriulli et al. (2008) [20]	Italy	Randomized, double-blind, multicenter superiority trial	NCT00374101	Patients with high-risk bleeding peptic ulcers after successful endoscopic hemostasis	482 randomized (243/239); 474 analyzed (238/236)	66.3 ± 15.6/66.8 ± 16.7
Yüksel et al. (2008) [21]	Turkey	Prospective, randomized, single-center trial	NR	Patients with active bleeding or a nonbleeding visible vessel after successful combined endoscopic hemostasis	99 randomized (50/49); 97 analyzed (48/49)	60.43 ± 14.02/56.28 ± 17.58
Mesihović et al. (2009) [22]	Bosnia and Herzegovina	Randomized, single-center trial	NR	Patients with high-risk bleeding peptic ulcers after successful endoscopic hemostasis	69 (34/35)	55.0 ± 16.9/62.3 ± 17.9
Choi et al. (2009) [23]	South Korea	Randomized, single-center, three-arm trial	NR	Patients aged ≥16 years with benign bleeding gastric or duodenal ulcers after endoscopic hemostasis	61 (40/21)	61.0 ± 16.5 and 57.4 ± 14.5/60.6 ± 11.5
Hsu et al. (2010) [24]	Taiwan	Open-label, randomized, single-center controlled trial	NCT00731601	Adults with high-risk bleeding peptic ulcers after successful endoscopic hemostasis	120 (60/60)	65.5/64.5; SD NR
Chan et al. (2011) [25]	Singapore	Prospective, randomized, double-blind, single-center equivalence trial	NCT00519519	Patients aged >21 years with high-risk bleeding peptic ulcers after successful endoscopic hemostasis	126 randomized (63/63); 122 analyzed (61/61)	63.5 ± 14.7/64.2 ± 13.1
Yamada and Wongwanakul (2012) [26]	Thailand	Prospective, block-randomized, single-center controlled trial	NR	Patients with massive bleeding peptic ulcers after successful therapeutic endoscopy	28 (15/13)	63.8 ± 14.6/56.5 ± 12.3
Chen et al. (2012) [27]	Taiwan	Prospective, randomized, single-center superiority trial	NCT00709046	Patients aged ≥16 years with high-risk bleeding peptic ulcers after successful combined endoscopic hemostasis	201 (100/101)	65.5 ± 15.1/64.9 ± 12.2
Chiu et al. (2016) [18]	Hong Kong	Prospective, randomized, single-center noninferiority trial	NCT00164931	Patients aged 15–90 years with high-risk bleeding peptic ulcers after successful endoscopic hemostasis	305 (153/152)	67.1/67.4; SD NR
Jafari et al. (2024) [19]	Iran	Randomized, single-center clinical trial	IRCT20190518037478N2	Adults aged 18–80 years with high-risk upper gastrointestinal ulcer bleeding after endoscopic therapy	80 (40/40)	52.87 ± 13.78/48.87 ± 13.68
(**B**)						
**Study**	**Ulcer Location**		**Forrest** **Classification**	**Endoscopic Management**		**PPI**
Hung et al. (2007) [16]	Gastric or duodenal		Ia, Ib, IIa, IIb	Diluted epinephrine injection with heat-probe coagulation when indicated		Pantoprazole
Garrido et al. (2008) [17]	Gastric or duodenal		Ia, Ib, IIa	Urgent endoscopy; endoscopic therapy in 90% and 100% of the respective groups using diluted epinephrine (1:10,000) plus 2% polidocanol; routine endoscopic reassessment at 24–48 h		Pantoprazole
Andriulli et al. (2008) [20]	Gastric or duodenal		Ia, Ib, IIa, IIb	Epinephrine injection alone or combined with thermal or mechanical therapy; selective repeat endoscopy in high-risk patients		Omeprazole or pantoprazole
Yüksel et al. (2008) [21]	Gastric or duodenal		Ia, Ib, IIa	Combined diluted epinephrine injection and heater-probe coagulation		Pantoprazole
Mesihović et al. (2009) [22]	Gastric or duodenal		Ia, Ib, IIa, IIb	Epinephrine injection alone or combined with hemoclips		Pantoprazole
Choi et al. (2009) [23]	Gastric or duodenal		Ia, Ib, IIa, IIb	Diluted epinephrine injection, argon plasma coagulation, and/or hemoclips		Pantoprazole
Hsu et al. (2010) [24]	Gastric or duodenal		Ia, Ib, IIa, IIb	Multipolar electrocoagulation with optional diluted epinephrine injection		Pantoprazole
Chan et al. (2011) [25]	Gastric, duodenal, or stomal		Ia, Ib, IIa, IIb	Diluted adrenaline injection alone or combined with heater-probe coagulation or hemoclips		Omeprazole
Yamada and Wongwanakul (2012) [26]	Gastric or duodenal		Ia, Ib, IIa, IIb	Saline–adrenaline injection alone or combined with bipolar electrocoagulation or hemoclips		Pantoprazole
Chen et al. (2012) [27]	Gastric or duodenal		Ia, Ib, IIa	Combined diluted epinephrine injection and heater-probe thermocoagulation		Pantoprazole
Chiu et al. (2016) [18]	Gastric, duodenal, or anastomotic		Ia, Ib, IIa, IIb	Epinephrine injection followed by heat-probe coagulation or hemoclips; scheduled second-look endoscopy at 16–24 h in the bolus group		Omeprazole
Jafari et al. (2024) [19]	NR		IIa, IIb	Adrenaline injection or thermal therapy using bipolar electrocoagulation or a heater probe; repeat endoscopy at 72 h		Pantoprazole

Note: Continuous/bolus indicates participants assigned to continuous intravenous PPI infusion and intermittent intravenous bolus therapy, respectively. Sample sizes refer to participants randomized to the eligible comparison arms; analyzed counts are shown when post-randomization exclusions occurred. Hung et al. also included a no-treatment arm, which was not eligible for this review. Choi et al. included two continuous-infusion arms (80-mg loading dose followed by 8 mg/h and 40-mg loading dose followed by 4 mg/h); their ages are shown separately before the slash. Across the eligible arms, 1726 participants were randomized: 876 to continuous infusion and 850 to intermittent bolus therapy. NR, not reported; PPI, proton pump inhibitor; SD, standard deviation. In Jafari et al., Forrest categories were derived from the reported endoscopic stigmata (nonbleeding visible vessel and adherent clot). In Chiu et al., the intermittent regimen was accompanied by scheduled second-look endoscopy and retreatment.

**Table 2:** (**A**) Concise comparison of intravenous proton pump inhibitor regimens across the included randomized controlled trials. (**B**) Outcome-assessment periods and reported outcomes of the included randomized controlled trials.

(**A**)					
**Study**	**PPI**	**Continuous-Infusion Regimen**	**Intermittent Regimen**	Intermittent Daily Dose *	**IV Treatment Duration**
Hung et al. (2007) [16]	Pantoprazole	80-mg IV loading dose, followed by 8 mg/h	80-mg IV loading dose, followed by 40 mg every 12 h	80 mg/day	72 h
Garrido et al. (2008) [17]	Pantoprazole	80-mg IV loading dose, followed by 8 mg/h	40 mg IV once daily	40 mg/day	72 h
Andriulli et al. (2008) [20]	Omeprazole or pantoprazole	80-mg IV loading dose, followed by 8 mg/h	40 mg IV once daily	40 mg/day	72 h
Yüksel et al. (2008) [21]	Pantoprazole	80-mg IV loading dose, followed by 8 mg/h	40 mg IV every 12 h	80 mg/day	72 h
Mesihović et al. (2009) [22]	Pantoprazole	8 mg/h; separate loading dose not clearly reported	40 mg IV twice daily	80 mg/day	3 days
Choi et al. (2009) [23]	Pantoprazole	Two arms: 80-mg loading dose followed by 8 mg/h, or 40-mg loading dose followed by 4 mg/h	40 mg IV once daily	40 mg/day	72 h
Hsu et al. (2010) [24]	Pantoprazole	Common 80-mg IV loading dose, followed by 8 mg/h	Common 80-mg IV loading dose, followed by 40 mg every 6 h	160 mg/day	72 h
Chan et al. (2011) [25]	Omeprazole	80-mg IV loading dose, followed by 8 mg/h	40 mg IV once daily	40 mg/day	72 h
Yamada and Wongwanakul (2012) [26]	Pantoprazole	Common 80-mg IV loading dose; 8 mg/h on days 1–3, followed by 40 mg every 12 h on days 4–7	Common 80-mg IV loading dose, followed by 40 mg every 12 h	80 mg/day	7 days
Chen et al. (2012) [27]	Pantoprazole	80-mg IV loading dose, followed by 8 mg/h	40 mg IV once daily	40 mg/day	72 h
Chiu et al. (2016) [18]	Omeprazole	80-mg IV loading dose, followed by 8 mg/h	40 mg IV every 12 h	80 mg/day	72 h
Jafari et al. (2024) [19]	Pantoprazole	8 mg/h; separate loading dose not reported	40 mg IV every 8 h	120 mg/day	3 days
(**B**)					
**Study**	**Outcome Assessment**		**Reported Outcomes**		
Hung et al. (2007) [16]	Primary clinical endpoint: rebleeding within 30 days; gastric pH monitored for 24 h; healing endoscopy at 8 weeks		Rebleeding; operative intervention; packed-cell transfusion; length of hospital stay; mortality; intragastric pH; adverse effects; ulcer healing		
Garrido et al. (2008) [17]	Index hospitalization; early endoscopic reassessment at 24–48 h		Persistent or recurrent bleeding; patients receiving transfusion; surgery; mortality		
Andriulli et al. (2008) [20]	Primary endpoint: in-hospital rebleeding; time categories of ≤24 h and >24 h to 7 days also reported		Rebleeding overall and by timing, Forrest class, ulcer location, Rockall score, endoscopic modality, and PPI; blood units transfused; patients receiving ≥2 units; hospital stay; surgery; bleeding-related and all-cause mortality		
Yüksel et al. (2008) [21]	Primary endpoint: early rebleeding within 72 h; outcomes assessed during the index hospitalization		Rebleeding; rescue endoscopic hemostasis; re-rebleeding; urgent surgery; red-blood-cell transfusion; hospital stay; bleeding-related and cardiac mortality; adverse effects		
Mesihović et al. (2009) [22]	Primary endpoint: in-hospital rebleeding; 24-h and 7-day data reported		Overall, 24-h, and 7-day rebleeding; gastric- and duodenal-ulcer rebleeding; surgery; mortality; transfusion requirements; hospital stay		
Choi et al. (2009) [23]	Primary pharmacodynamic assessment: 24-h intragastric pH; rebleeding assessed through 30 days		Intragastric pH parameters; 30-day rebleeding; urgent endoscopic reassessment and rescue therapy; pantoprazole pharmacokinetics; associations with CYP2C19 genotype and H. pylori status		
Hsu et al. (2010) [24]	Primary endpoint: recurrent bleeding within 14 days		Recurrent bleeding; blood-transfusion volume; hospital stay; surgery; all-cause mortality		
Chan et al. (2011) [25]	Primary endpoint: recurrent bleeding within 30 days; final clinical evaluation at 4 weeks		Rebleeding; endoscopic retreatment; surgery; blood units transfused; hospital stay; mortality; adverse effects		
Yamada and Wongwanakul (2012) [26]	Primary endpoint: recurrent bleeding within 7 days		Overall and 7-day rebleeding; gastric- and duodenal-ulcer rebleeding; surgery; mortality; blood units transfused; hospital stay		
Chen et al. (2012) [27]	Primary endpoint: recurrent bleeding within 30 days; 72-h and 7-day data also reported		Rebleeding at 72 h, 7 days, and 30 days; blood units transfused; hospital stay; surgery or transcatheter arterial embolization; all-cause and bleeding-related mortality; predictors of rebleeding		
Chiu et al. (2016) [18]	Primary endpoint: recurrent bleeding within 30 days		Rebleeding; surgery; blood-transfusion volume; intensive-care-unit and hospital stay; morbidity; 30-day mortality		
Jafari et al. (2024) [19]	Primary assessment: day-3 endoscopic appearance and treatment failure		Change from high-risk to low-risk ulcer stigmata; persistent or recurrent bleeding at day 3; packed-cell transfusion; hospital stay; urgent surgery; mortality		

Note: * Excludes any initial loading dose. Hsu et al. and Yamada and Wongwanakul administered a common 80-mg loading dose to both groups; Hung et al. also used an 80-mg loading dose in the intermittent group. Choi et al. evaluated two continuous-infusion arms. Saline placebo used to maintain blinding is omitted. In Chiu et al., intermittent therapy included scheduled second-look endoscopy and retreatment. IV, intravenous; PPI, proton pump inhibitor. Note: Outcome assessment indicates the principal clinical or pharmacodynamic assessment period reported by each trial. In Jafari et al., treatment failure was determined from scheduled day-3 endoscopic appearance, including persistent or recurrent high-risk bleeding stigmata. Reported outcomes indicate availability in the source report and do not imply eligibility for quantitative synthesis. CYP2C19, cytochrome P450 2C19; H. pylori, Helicobacter pylori; IV, intravenous; PPI, proton pump inhibitor.

**Table 3:** (**A**) Dichotomous data available for the primary, time-specific, subgroup, and secondary quantitative syntheses. (**B**) Study-level continuous data available for the secondary quantitative syntheses.

(**A**)					
**Outcome**	**Contributing Studies**	**No. of Studies**	**Continuous** **Infusion**	**Intermittent** **Regimen**	**Effect Measure**
Primary quantitative synthesis					
Clinical ulcer rebleeding (overall)	Hung et al. [16]; Garrido et al. [17]; Andriulli et al. [20]; Yüksel et al. [21]; Mesihović et al. [22]; Choi et al. [23]; Hsu et al. [24]; Chan et al. [25]; Yamada and Wongwanakul [26]; Chen et al. [27]; Chiu et al. [18]	11	72/823	76/798	RR
Time-specific quantitative syntheses					
Clinical ulcer rebleeding within 24 h	Andriulli et al. [20]; Mesihović et al. [22]; Chiu et al. [18]	3	16/425	19/423	RR
Clinical ulcer rebleeding within 7 days	Andriulli et al. [20]; Mesihović et al. [22]; Choi et al. [23]; Yamada and Wongwanakul [26]; Chen et al. [27]; Chiu et al. [18]	6	55/580	50/558	RR
Exploratory time-point quantitative synthesis					
Clinical ulcer rebleeding within 72 h	Andriulli et al. [20]; Yüksel et al. [21]; Choi et al. [23]; Yamada and Wongwanakul [26]; Chen et al. [27]	5	31/441	32/420	RR
Prespecified subgroup quantitative syntheses					
Clinical ulcer rebleeding in gastric ulcers	Andriulli et al. [20]; Mesihović et al. [22]; Yamada and Wongwanakul [26]	3	18/124	17/131	RR
Clinical ulcer rebleeding in duodenal ulcers	Andriulli et al. [20]; Mesihović et al. [22]; Yamada and Wongwanakul [26]	3	20/162	14/152	RR
Secondary quantitative syntheses					
Need for surgery	Hung et al. [16]; Garrido et al. [17]; Andriulli et al. [20]; Yüksel et al. [21]; Mesihović et al. [22]; Hsu et al. [24]; Chan et al. [25]; Yamada and Wongwanakul [26]; Chen et al. [27]; Chiu et al. [18]; Jafari et al. [19]	11	24/820	16/812	RR
All-cause mortality	Hung et al. [16]; Garrido et al. [17]; Andriulli et al. [20]; Yüksel et al. [21]; Mesihović et al. [22]; Hsu et al. [24]; Chan et al. [25]; Yamada and Wongwanakul [26]; Chen et al. [27]; Chiu et al. [18]; Jafari et al. [19]	11	21/822	11/812	RR
Patients meeting the study-defined transfusion threshold	Garrido et al. [17]; Andriulli et al. [20]; Yüksel et al. [21]; Mesihović et al. [22]; Jafari et al. [19]	5	190/380	168/381	RR
(**B**)					
**Outcome**	**Study**	**Continuous Infusion**	**Intermittent Regimen**		**Effect Measure**
Blood units transfused	Andriulli et al. [20]	1.70 ± 2.10	1.50 ± 2.10		MD
	Yüksel et al. [21]	2.59 ± 2.40	2.18 ± 1.89		MD
	Mesihović et al. [22]	2.18 ± 0.80	1.34 ± 1.02		MD
	Chan et al. [25]	2.10 ± 1.70	2.40 ± 2.00		MD
	Yamada and Wongwanakul [26]	6.07 ± 3.80	6.31 ± 3.80		MD
	Chen et al. [27]	2.90 ± 6.20	1.60 ± 2.20		MD
	Chiu et al. [18]	2.20 ± 2.70	1.90 ± 2.40		MD
Hospital stay, days	Andriulli et al. [20]	7.30 ± 4.40	6.60 ± 3.70		MD
	Yüksel et al. [21]	4.41 ± 1.82	4.17 ± 1.72		MD
	Mesihović et al. [22]	5.80 ± 2.80	6.40 ± 2.80		MD
	Chan et al. [25]	5.20 ± 2.10	5.90 ± 6.40		MD
	Yamada and Wongwanakul [26]	9.93 ± 4.30	10.15 ± 3.20		MD
	Chen et al. [27]	7.10 ± 7.80	5.90 ± 3.30		MD
	Jafari et al. [19]	5.45 ± 2.55	4.92 ± 0.92		MD

Note: Data are presented as events/total participants. The number of studies indicates the trials contributing usable data to each outcome, including trials with zero events in both treatment groups. Rebleeding within 72 h was evaluated as an exploratory analysis using a harmonized time window. For Choi et al. events and participants from the two continuous-infusion arms were summed for each applicable rebleeding time point, whereas the common intermittent comparator was included only once; no continuous outcome from this trial contributed to a quantitative synthesis. Jafari et al. assessed treatment failure based on scheduled day-3 endoscopy rather than clinically detected rebleeding and was therefore excluded from the primary clinical-rebleeding synthesis. Definitions of transfusion requirements varied across studies: Garrido et al., Yüksel et al., and Jafari et al. reported receipt of any transfusion, whereas Andriulli et al. and Mesihović et al. reported receipt of at least two units. Therefore, this exploratory synthesis should be interpreted cautiously. Transcatheter arterial embolization was not quantitatively synthesized because fewer than two trials reported comparable data. RR, risk ratio. Continuous data are presented as mean ± standard deviation. Seven trials (1288 participants) contributed to the synthesis of blood units transfused, and seven trials (1063 participants) contributed to the synthesis of hospital stay. Transfusion data for the Mesihović trial were obtained from the complementary publication by Mehmedović-Redžepović et al., which reported the same cohort and treatment groups; the trial was counted once. Hung et al. reported means without standard deviations, Hsu et al. reported transfusion volume in milliliters and hospital stay without standard deviations, and Chiu et al. reported hospital stay as median (range); these data were not combined in the corresponding continuous syntheses. MD, mean difference.

**Table 4:** Summary of findings for continuous infusion compared with intermittent bolus proton pump inhibitor therapy following endoscopic hemostasis.

Outcomes	Risk with Intermittent Bolus	Risk with Continuous Infusion (95% CI)	Relative Effect (95% CI)	Participants (Studies)	Certainty of the Evidence (GRADE)	Interpretation
Overall ulcer rebleeding	95 per 1000	89 per 1000 (62–129)	RR 0.93 (0.65–1.35)	1621 (11 RCTs)	⊕◯◯◯ Very low ^a,b,c^	The evidence is very uncertain about the effect on overall rebleeding.
Ulcer rebleeding within 24 h	45 per 1000	31 per 1000 (4–253)	RR 0.70 (0.09–5.64)	848 (3 RCTs)	⊕◯◯◯ Very low ^a,b^	The evidence is very uncertain about the effect on rebleeding within 24 h.
Ulcer rebleeding within 7 days	90 per 1000	96 per 1000 (59–154)	RR 1.07 (0.66–1.72)	1138 (6 RCTs)	⊕◯◯◯ Very low ^a,b^	The evidence is very uncertain about the effect on rebleeding within 7 days.
Need for surgical intervention	20 per 1000	28 per 1000 (16–50)	RR 1.42 (0.80–2.53)	1632 (11 RCTs)	⊕⊕◯◯ Low ^a,d^	Continuous infusion may result in little or no difference, but clinically important harm cannot be excluded.
All-cause mortality	14 per 1000	24 per 1000 (12–47)	RR 1.76 (0.90–3.44)	1634 (11 RCTs)	⊕⊕◯◯ Low ^a,d^	Continuous infusion may result in little or no difference, but clinically important harm cannot be excluded.
Mean number of blood units transfused	—	MD 0.37 units higher (0.06 lower to 0.80 higher)	—	1288 (7 RCTs)	⊕⊕⊕◯ Moderate ^a^	Continuous infusion probably results in little or no clinically important difference in transfusion requirements.
Length of hospital stay	—	MD 0.35 days longer (0.12 shorter to 0.82 longer)	—	1063 (7 RCTs)	⊕⊕⊕◯ Moderate ^a^	Continuous infusion probably results in little or no clinically important difference in hospital stay.

Note: Population: Predominantly adult patients with bleeding peptic ulcers, with randomization occurring after successful endoscopic hemostasis in most trials. Intervention: Continuous intravenous proton pump inhibitor infusion. Comparator: Intermittent intravenous proton pump inhibitor bolus therapy. Abbreviations: CI, confidence interval; GRADE, Grading of Recommendations Assessment, Development and Evaluation; MD, mean difference; RCT, randomized controlled trial; RR, risk ratio. GRADE: high certainty, ⊕⊕⊕◯; low certainty, ⊕⊕◯◯; very low certainty, ⊕◯◯◯. ^a^ Downgraded for risk of bias. ^b^ Downgraded for very serious imprecision. ^c^ Downgraded for suspected publication bias. ^d^ Downgraded for serious imprecision. Detailed outcome-specific judgments and explanations are provided in Supplementary Table S4.

## Data Availability

All data generated or analyzed during this systematic review and meta-analysis are contained within the article and its Supplementary Materials.

## Supplementary Materials

The following supporting information can be downloaded at https://www.mdpi.com/article/10.3390/jcm15197743/s1: Table S1, Complete search strategies used in the electronic databases; Table S2, Outcome-specific risk-of-bias assessment of the included randomized controlled trials using RoB 2; Table S3, Sensitivity analyses and common-effect Mantel–Haenszel analyses; Table S4, GRADE assessment of the certainty of evidence for the seven outcomes included in the Summary of Findings table. Table S5, PRISMA 2020 checklist.

## Abbreviations

The following abbreviations are used in this manuscript:

CI	confidence interval
CYP2C19	cytochrome P450 family 2 subfamily C member 19
GRADE	Grading of Recommendations Assessment, Development and Evaluation
H. pylori	Helicobacter pylori
I^2^	I-squared statistic
IV	intravenous
MD	mean difference
NR	not reported
OIS	optimal information size
OSF	Open Science Framework
pH	measure of acidity or alkalinity
PICOS	Population, Intervention, Comparator, Outcomes, and Study design
PPI	proton pump inhibitor
PRISMA	Preferred Reporting Items for Systematic Reviews and Meta-Analyses
RCT	randomized controlled trial
REML	restricted maximum likelihood
RoB 2	revised Cochrane risk-of-bias tool for randomized trials
RR	risk ratio
SD	standard deviation
SE	standard error
SoF	Summary of Findings
τ^2^	tau-squared between-study variance
