# Supplementary material for: Continuous Infusion Versus Intermittent Bolus Proton Pump Inhibitor Therapy Following Endoscopic Hemostasis for Bleeding Peptic Ulcers: A Systematic Review and Meta-Analysis
Source: J Clin Med. 2026 Oct 8;15(19):7743. doi: 10.3390/jcm15197743 (PMC13666069; doi:10.3390/jcm15197743)
Supplement: Supplementary file 1 [file jcm-15-07743-s001.zip › Supplementary_Table_S1.pdf]

Supplementary Materials

Continuous Infusion versus Intermittent Bolus Proton Pump Inhibitor Therapy Following Endoscopic Hemostasis for Bleeding Peptic Ulcers: A Systematic Review and Meta-analysis

Table S1. Complete search strategies used in the electronic databases.

PubMed

Platform: NCBI PubMed **Search date:** 29 June 2026 **Filters or limits:** None **Records retrieved:** n = 388

Search field and syntax: Standard PubMed search interface; no field tags were applied. Terms were informed by MeSH and DeCS.

Exact search strategy

S1: "Inhibitor, Proton Pump" OR "Inhibitors, Proton Pump" OR "Proton Pump Inhibitor" OR "Proton Pump Inhibitors" OR "Pump Inhibitor, Proton"

S2: "Gastroduodenal Ulcer" OR "Gastroduodenal Ulcers" OR "Marginal Ulcer" OR "Marginal Ulcers" OR "Peptic Ulcer" OR "Peptic Ulcers" OR "Ulcer, Gastroduodenal" OR "Ulcer, Marginal" OR "Ulcer, Peptic" OR "Ulcers, Gastroduodenal" OR "Ulcers, Marginal" OR "Ulcers, Peptic"

S3: "Randomized Controlled Trial" OR "Randomised Controlled Trial" OR "Clinical Trials, Randomized" OR "Trials, Randomized Clinical" OR "Controlled Clinical Trials, Randomized" OR "controlled clinical trial" OR "clinical trial"

FS: (S1) AND (S2) AND (S3)

Scopus

Platform: Elsevier Scopus **Search date:** 29 June 2026 **Filters or limits:** None **Records retrieved:** n = 2030

Search field and syntax: Title, abstract, and author keywords (TITLE-ABS-KEY). Terms were informed by MeSH and DeCS.

Exact search strategy

S1: TITLE-ABS-KEY ("Inhibitor, Proton Pump" OR "Inhibitors, Proton Pump" OR "Proton Pump Inhibitor" OR "Proton Pump Inhibitors" OR "Pump Inhibitor, Proton")

S2: TITLE-ABS-KEY ("Gastroduodenal Ulcer" OR "Gastroduodenal Ulcers" OR "Marginal Ulcer" OR "Marginal Ulcers" OR "Peptic Ulcer" OR "Peptic Ulcers" OR "Ulcer, Gastroduodenal" OR "Ulcer, Marginal" OR "Ulcer, Peptic" OR "Ulcers, Gastroduodenal" OR "Ulcers, Marginal" OR "Ulcers, Peptic")

S3: TITLE-ABS-KEY ("Randomized Controlled Trial" OR "Randomised Controlled Trial" OR "Clinical Trials, Randomized" OR "Trials, Randomized Clinical" OR "Controlled Clinical Trials, Randomized" OR "controlled clinical trial" OR "clinical trial")

FS: (S1) AND (S2) AND (S3)

Web of Science

Platform: Clarivate Web of Science **Search date:** 29 June 2026 **Filters or limits:** None **Records retrieved:** n = 420

Search field and syntax: Standard Web of Science search interface; no field tags were applied. Terms were informed by MeSH and DeCS.

Exact search strategy

S1: "Inhibitor, Proton Pump" OR "Inhibitors, Proton Pump" OR "Proton Pump Inhibitor" OR "Proton Pump Inhibitors" OR "Pump Inhibitor, Proton"

S2: "Gastroduodenal Ulcer" OR "Gastroduodenal Ulcers" OR "Marginal Ulcer" OR "Marginal Ulcers" OR "Peptic Ulcer" OR "Peptic Ulcers" OR "Ulcer, Gastroduodenal" OR "Ulcer, Marginal" OR "Ulcer, Peptic" OR "Ulcers, Gastroduodenal" OR "Ulcers, Marginal" OR "Ulcers, Peptic"

S3: "Randomized Controlled Trial" OR "Randomised Controlled Trial" OR "Clinical Trials, Randomized" OR "Trials, Randomized Clinical" OR "Controlled Clinical Trials, Randomized" OR "controlled clinical trial" OR "clinical trial"

FS: (S1) AND (S2) AND (S3)

ScienceDirect

Platform: Elsevier ScienceDirect **Search date:** 29 June 2026 **Filters or limits:** None **Records retrieved:** n = 2230

Search field and syntax: Find articles with these terms (all document fields excluding references). A simplified three-concept query was used because of interface constraints.

Exact search strategy

S1: "Proton Pump Inhibitors"

S2: "Peptic Ulcer"

S3: "Randomized Controlled Trial"

FS: ("Proton Pump Inhibitors") AND ("Peptic Ulcer") AND ("Randomized Controlled Trial")

Embase

Platform: Elsevier Embase.com **Search date:** 29 June 2026 **Filters or limits:** None **Records retrieved:** n = 1504

Search field and syntax: Standard Embase search interface; no field tags or /exp commands were applied. Terms were informed by Emtree.

Exact search strategy

S1: 'gastric proton pump inhibitor' OR 'hydrogen potassium adenosine triphosphatase inhibitor' OR 'hydrogen potassium atpase inhibitor' OR 'proton pump inhibitors' OR 'proton pump inhibitor'

S2: 'acid peptic disease' OR 'acute peptic ulcer' OR 'chronic peptic ulcer' OR 'gastro duodenal ulcus' OR 'gastroduodenal ulcer' OR 'peptic ulcer disease' OR 'peptic ulceration' OR 'peptic ulcus' OR 'peptic, ulcus' OR 'ulcer, gastroduodenal' OR 'ulcer, peptic' OR 'ulcus gastro duodenalis' OR 'ulcus pepticum' OR 'ulcus, pepticum' OR 'peptic ulcer'

S3: 'controlled trial, randomized' OR 'randomised controlled study' OR 'randomised controlled trial' OR 'randomized controlled study' OR 'trial, randomized controlled' OR 'randomized controlled trial'

FS: (S1) AND (S2) AND (S3)

Abbreviations: DeCS, Health Sciences Descriptors; FS, final search strategy; MeSH, Medical Subject Headings; S, search set. Searches covered each electronic source from inception to 29 June 2026. No restrictions on publication year or language and no other filters or limits were applied.
