# Supplementary material for: Continuous Infusion Versus Intermittent Bolus Proton Pump Inhibitor Therapy Following Endoscopic Hemostasis for Bleeding Peptic Ulcers: A Systematic Review and Meta-Analysis
Source: J Clin Med. 2026 Oct 8;15(19):7743. doi: 10.3390/jcm15197743 (PMC13666069; doi:10.3390/jcm15197743)
Supplement: Supplementary file 1 [file jcm-15-07743-s001.zip › Supplementary_Table_S2.pdf]

Supplementary Materials

Continuous Infusion versus Intermittent Bolus Proton Pump Inhibitor Therapy Following Endoscopic Hemostasis for Bleeding Peptic Ulcers: A Systematic Review and Meta-analysis

Table S2. Outcome-specific risk-of-bias assessment of the included randomized controlled trials using RoB 2.

Study	Outcome	D1	D2	D3	D4	D5	Overall risk of bias
**Hung et al. (2007)**	Overall ulcer rebleeding (longest reported follow-up)	Some concerns	High	High	Some concerns	Some concerns	High
Hung et al. (2007)	Need for surgical intervention	Some concerns	High	High	Some concerns	Some concerns	High
Hung et al. (2007)	All-cause mortality	Some concerns	High	High	Low	Some concerns	High
**Garrido et al. (2008)**	Overall ulcer rebleeding (longest reported follow-up)	Some concerns	Low	Low	Some concerns	Some concerns	Some concerns
Garrido et al. (2008)	Need for surgical intervention	Some concerns	Low	Low	Some concerns	Some concerns	Some concerns
Garrido et al. (2008)	All-cause mortality	Some concerns	Low	Low	Low	Some concerns	Some concerns
**Andriulli et al. (2008)**	Overall ulcer rebleeding (longest reported follow-up)	Some concerns	Low	Low	Low	Some concerns	Some concerns
Andriulli et al. (2008)	Rebleeding within 24 h	Some concerns	Low	Some concerns	Low	Some concerns	Some concerns
Andriulli et al. (2008)	Rebleeding within 7 days	Some concerns	Low	Low	Low	Some concerns	Some concerns
Andriulli et al. (2008)	Need for surgical intervention	Some concerns	Low	Some concerns	Low	Some concerns	Some concerns
Andriulli et al. (2008)	All-cause mortality	Some concerns	Low	Some concerns	Low	Some concerns	Some concerns
Andriulli et al. (2008)	Mean number of blood units transfused	Some concerns	Low	Low	Low	Some concerns	Some concerns
Andriulli et al. (2008)	Length of hospital stay	Some concerns	Low	Low	Low	Some concerns	Some concerns
**Yüksel et al. (2008)**	Overall ulcer rebleeding (72 h)	Some concerns	High	Some concerns	Low	Some concerns	High
Yüksel et al. (2008)	Need for surgical intervention	Some concerns	High	Some concerns	Low	Some concerns	High
Yüksel et al. (2008)	All-cause mortality	Some concerns	Low	Low	Low	Some concerns	Some concerns
Yüksel et al. (2008)	Mean number of blood units transfused	Some concerns	Some concerns	Low	Low	Some concerns	Some concerns
Yüksel et al. (2008)	Length of hospital stay	Some concerns	Some concerns	Low	Low	Some concerns	Some concerns
**Mesihović et al. (2009)**	Overall ulcer rebleeding (longest reported follow-up)	Some concerns	Low	Low	Some concerns	Some concerns	Some concerns
Mesihović et al. (2009)	Rebleeding within 24 h	Some concerns	Low	Low	Some concerns	Some concerns	Some concerns
Mesihović et al. (2009)	Rebleeding within 7 days	Some concerns	Low	Low	Some concerns	Some concerns	Some concerns
Mesihović et al. (2009)	Need for surgical intervention	Some concerns	Low	Low	Some concerns	Some concerns	Some concerns
Mesihović et al. (2009)	All-cause mortality	Some concerns	Low	Low	Low	Some concerns	Some concerns
Mesihović et al. (2009)	Mean number of blood units transfused	Some concerns	Low	Low	Some concerns	Some concerns	Some concerns
Mesihović et al. (2009)	Length of hospital stay	Some concerns	Low	Low	Some concerns	Some concerns	Some concerns

Study	Outcome	D1	D2	D3	D4	D5	Overall risk of bias
**Choi et al. (2009)**	Overall ulcer rebleeding (longest reported follow-up)	Some concerns	Low	Low	Some concerns	Some concerns	Some concerns
Choi et al. (2009)	Rebleeding within 7 days	Some concerns	Low	Low	Some concerns	Some concerns	Some concerns
**Hsu et al. (2010)**	Overall ulcer rebleeding (longest reported follow-up)	Low	Low	Low	Some concerns	Some concerns	Some concerns
Hsu et al. (2010)	Need for surgical intervention	Low	Low	Low	Some concerns	Some concerns	Some concerns
Hsu et al. (2010)	All-cause mortality	Low	Low	Low	Low	Some concerns	Some concerns
Hsu et al. (2010)	Mean number of blood units transfused	Low	Low	Low	Some concerns	Some concerns	Some concerns
Hsu et al. (2010)	Length of hospital stay	Low	Low	Low	Some concerns	Some concerns	Some concerns
**Chan et al. (2011)**	Overall ulcer rebleeding (longest reported follow-up)	Low	Low	Low	Low	Low	Low
Chan et al. (2011)	Need for surgical intervention	Low	Low	Low	Low	Low	Low
Chan et al. (2011)	All-cause mortality	Low	Low	Low	Low	Low	Low
Chan et al. (2011)	Mean number of blood units transfused	Low	Low	Low	Some concerns	Some concerns	Some concerns
Chan et al. (2011)	Length of hospital stay	Low	Low	Low	Low	Low	Low
**Yamada and Wongwanakul (2012)**	Overall ulcer rebleeding (longest reported follow-up)	Some concerns	Low	Low	Some concerns	Some concerns	Some concerns
Yamada and Wongwanakul (2012)	Rebleeding within 7 days	Some concerns	Low	Low	Some concerns	Some concerns	Some concerns
Yamada and Wongwanakul (2012)	Need for surgical intervention	Some concerns	Low	Low	Some concerns	Some concerns	Some concerns
Yamada and Wongwanakul (2012)	All-cause mortality	Some concerns	Low	Low	Low	Some concerns	Some concerns
Yamada and Wongwanakul (2012)	Mean number of blood units transfused	Some concerns	Low	Low	Some concerns	Some concerns	Some concerns
Yamada and Wongwanakul (2012)	Length of hospital stay	Some concerns	Low	Low	Some concerns	Some concerns	Some concerns
**Chen et al. (2012)**	Overall ulcer rebleeding (longest reported follow-up)	Low	Low	Low	Some concerns	Some concerns	Some concerns
Chen et al. (2012)	Rebleeding within 7 days	Low	Low	Low	Some concerns	Some concerns	Some concerns
Chen et al. (2012)	Need for surgical intervention	Low	Low	Low	Some concerns	Some concerns	Some concerns
Chen et al. (2012)	All-cause mortality	Low	Low	Low	Low	Some concerns	Some concerns
Chen et al. (2012)	Mean number of blood units transfused	Low	Low	Low	Some concerns	Some concerns	Some concerns
Chen et al. (2012)	Length of hospital stay	Low	Low	Low	Some concerns	Some concerns	Some concerns

Study	Outcome	D1	D2	D3	D4	D5	Overall risk of bias
**Chiu et al. (2016)**	Overall ulcer rebleeding (longest reported follow-up)	Low	Low	Low	Some concerns	Some concerns	Some concerns
Chiu et al. (2016)	Rebleeding within 24 h	Low	Low	Low	Some concerns	Some concerns	Some concerns
Chiu et al. (2016)	Rebleeding within 7 days	Low	Low	Low	Some concerns	Some concerns	Some concerns
Chiu et al. (2016)	Need for surgical intervention	Low	Low	Low	Some concerns	Some concerns	Some concerns
Chiu et al. (2016)	All-cause mortality	Low	Low	Low	Low	Some concerns	Some concerns
Chiu et al. (2016)	Mean number of blood units transfused	Low	Low	Low	Some concerns	Some concerns	Some concerns
Chiu et al. (2016)	Length of hospital stay	Low	Low	Low	Some concerns	Some concerns	Some concerns
**Jafari et al. (2024)**	Overall ulcer rebleeding (not reported)	N/A	N/A	N/A	N/A	N/A	N/A
Jafari et al. (2024)	Need for surgical intervention	Some concerns	Low	Low	Some concerns	Some concerns	Some concerns
Jafari et al. (2024)	All-cause mortality	Some concerns	Low	Low	Low	Some concerns	Some concerns
Jafari et al. (2024)	Patients receiving blood transfusion	Some concerns	Low	Low	Some concerns	Some concerns	Some concerns
Jafari et al. (2024)	Length of hospital stay	Some concerns	Low	Low	Some concerns	Some concerns	Some concerns

Note. RoB 2 judgments are specific to individual trial results. Overall ulcer rebleeding refers to the longest clinically reported follow-up available in each trial. The 72-h rebleeding result from Yüksel et al. (2008) was retained as that trial's longest available overall rebleeding assessment. For Chen et al. (2012), the rebleeding judgments correspond to the intention-to-treat estimates. Jafari et al. (2024) did not report a clinically defined rebleeding outcome compatible with the review and was therefore marked N/A for that result; its transfusion outcome represents receipt of any transfusion, not the mean number of blood units transfused. D1, bias arising from the randomization process; D2, bias due to deviations from intended interventions; D3, bias due to missing outcome data; D4, bias in measurement of the outcome; D5, bias in selection of the reported result. Low, low risk of bias; Some concerns, some concerns; High, high risk of bias; N/A, not applicable.
