# Supplementary material for: Continuous Infusion Versus Intermittent Bolus Proton Pump Inhibitor Therapy Following Endoscopic Hemostasis for Bleeding Peptic Ulcers: A Systematic Review and Meta-Analysis
Source: J Clin Med. 2026 Oct 8;15(19):7743. doi: 10.3390/jcm15197743 (PMC13666069; doi:10.3390/jcm15197743)
Supplement: Supplementary file 1 [file jcm-15-07743-s001.zip › Supplementary_Table_S3.pdf]

Supplementary Materials

Continuous Infusion versus Intermittent Bolus Proton Pump Inhibitor Therapy Following Endoscopic Hemostasis for Bleeding Peptic Ulcers: A Systematic Review and Meta-analysis

Table S3. Sensitivity analyses and common-effect Mantel-Haenszel analyses.

Outcome	Primary random-effects estimate (95% CI)	Trials (estimable)	Participants	Sensitivity estimate (95% CI)	p value	I^2 (%)	τ^2
**Common follow-up window: 30-day rebleeding**							
Overall rebleeding restricted to 30-day follow-up	RR 0.93 (0.65-1.35)	5	792	RR 0.64 (0.26-1.57)	0.241	20.0	0.057
**72-hour count discrepancy: Andriulli et al. excluded**							
Rebleeding within 72 hours	RR 0.96 (0.46-2.04)	4	387	RR 1.01 (0.20-5.00)	0.991	35.3	0
**Strict post-hemostasis framework: Garrido et al. excluded**							
Overall rebleeding	RR 0.93 (0.65-1.35)	10	1,580	RR 0.94 (0.63-1.41)	0.746	10.0	0.033
Need for surgery	RR 1.42 (0.80-2.53)	10 (9)	1,591	RR 1.45 (0.77-2.74)	0.216	0.0	0
All-cause mortality	RR 1.76 (0.90-3.44)	10 (7)	1,593	RR 1.71 (0.80-3.63)	0.133	0.0	0
Study-defined transfusion threshold	RR 1.11 (0.99-1.25)	4	720	RR 1.12 (0.97-1.30)	0.089	0.0	0
**Additional asymmetric co-intervention: Chiu et al. excluded**							
Overall rebleeding	RR 0.93 (0.65-1.35)	10	1,316	RR 0.93 (0.60-1.44)	0.725	9.8	0.054
Rebleeding within 24 hours	RR 0.70 (0.09-5.64)	2	543	RR 0.90 (0.001-768.87)	0.871	37.8	0.246
Rebleeding within 7 days	RR 1.07 (0.66-1.72)	5	833	RR 1.08 (0.57-2.07)	0.753	16.3	0.022
Rebleeding at 30 days	RR 0.64 (0.26-1.57)	4	487	RR 0.49 (0.11-2.17)	0.226	33.2	0.356
Need for surgery	RR 1.42 (0.80-2.53)	10 (9)	1,327	RR 1.30 (0.66-2.58)	0.400	0.0	0
All-cause mortality	RR 1.76 (0.90-3.44)	10 (7)	1,329	RR 1.48 (0.64-3.41)	0.291	0.0	0
Mean blood units transfused	MD 0.37 (-0.06 to 0.80)	6	983	MD 0.39 (-0.18 to 0.95)	0.138	56.2	0.156
**High overall risk of bias excluded**							
Overall rebleeding	RR 0.93 (0.65-1.35)	9	1,421	RR 0.89 (0.57-1.40)	0.581	19.3	0.055
**Combined exclusion: high overall risk of bias and asymmetric co-intervention**							
Overall rebleeding	RR 0.93 (0.65-1.35)	8	1,116	RR 0.87 (0.50-1.52)	0.581	28.4	0.103

Outcome	Primary random-effects estimate (95% CI)	Trials (estimable)	Participants	Sensitivity estimate (95% CI)	p value	I^2 (%)	τ^2
**Common-effect Mantel-Haenszel analyses**							
Overall rebleeding	RR 0.93 (0.65-1.35)	11	1,621	RR 0.92 (0.68-1.25)	0.608	1.4	-
Rebleeding within 24 hours	RR 0.70 (0.09-5.64)	3	848	RR 0.84 (0.44-1.61)	0.602	34.6	-
Rebleeding within 7 days	RR 1.07 (0.66-1.72)	6	1,138	RR 1.06 (0.74-1.52)	0.747	0.0	-
Exploratory rebleeding within 72 hours	RR 0.96 (0.46-2.04)	5	861	RR 0.92 (0.58-1.47)	0.736	14.5	-
**Rare-event sensitivity analyses: common-effect Mantel-Haenszel risk difference without continuity correction**							
Rebleeding within 72 hours	RR 0.96 (0.46-2.04)	5	861	RD -0.54 percentage points (-4.07 to 2.98)	0.763	-	-
Rebleeding in duodenal ulcers	RR 1.00 (0.28-3.55)	3	314	RD 2.69 percentage points (-3.97 to 9.36)	0.428	-	-
Need for surgery	RR 1.42 (0.80-2.53)	11	1,632	RD 0.92 percentage points (-0.53 to 2.36)	0.215	-	-
All-cause mortality	RR 1.76 (0.90-3.44)	11	1,634	RD 1.21 percentage points (-0.12 to 2.55)	0.075	-	-

Abbreviations: CI, confidence interval; MD, mean difference; RR, risk ratio. Primary estimates were obtained using random-effects models with restricted maximum-likelihood estimation and Hartung-Knapp confidence intervals. Exclusion analyses used the same model after removal of the indicated trial. Common-effect estimates were calculated using the Mantel-Haenszel method; tau-squared was not estimated for these analyses. The p values refer to the test of the pooled effect. Numbers in parentheses in the trials column indicate trials contributing estimable relative effects; double-zero trials were retained in participant totals but did not contribute to the pooled RR. The very wide confidence interval for rebleeding within 24 hours after exclusion of Chiu et al. reflects the Hartung-Knapp calculation with only two remaining trials. Garrido et al. did not contribute to the syntheses of mean blood units transfused or length of hospital stay. Chiu et al. did not contribute to the 72-hour, study-defined transfusion-threshold, or length-of-stay syntheses. The high-risk-of-bias analysis excluded Hung et al. and Yüksel et al.; the combined analysis also excluded Chiu et al. because of its asymmetric co-intervention. Risk-difference estimates are expressed as absolute percentage-point differences and were calculated as the risk in the continuous-infusion group minus the risk in the intermittent-bolus group using the original cell counts without continuity correction. Trials with zero events in both groups were retained in the surgery and mortality analyses. I-squared and tau-squared are not shown for these post hoc common-effect risk-difference analyses. None of the sensitivity analyses materially changed the interpretation of the findings.
