# Supplementary material for: Continuous Infusion Versus Intermittent Bolus Proton Pump Inhibitor Therapy Following Endoscopic Hemostasis for Bleeding Peptic Ulcers: A Systematic Review and Meta-Analysis
Source: J Clin Med. 2026 Oct 8;15(19):7743. doi: 10.3390/jcm15197743 (PMC13666069; doi:10.3390/jcm15197743)
Supplement: Supplementary file 1 [file jcm-15-07743-s001.zip › Supplementary_Table_S4.pdf]

Supplementary Materials

Continuous Infusion versus Intermittent Bolus Proton Pump Inhibitor Therapy Following Endoscopic Hemostasis for Bleeding Peptic Ulcers: A Systematic Review and Meta-analysis

Table S4. GRADE assessment of the certainty of evidence for the seven outcomes included in the Summary of Findings table.

Outcome	Studies (participants)	Risk of bias	Inconsistency	Indirectness	Imprecision	Publication bias	Relative effect (95% CI)	Absolute effect (95% CI)	Certainty of the evidence (GRADE)
**Overall ulcer rebleeding**	11 RCTs (n = 1,621)	Serious (−1)[a]	Not serious	Not serious	Very serious (−2)[a]	Strongly suspected (−1)[a]	RR 0.93 (0.65–1.35)	6 fewer per 1,000 (33 fewer to 34 more)	⊕◯◯◯ Very low
**Ulcer rebleeding within 24 h**	3 RCTs (n = 848)	Serious (−1)[b]	Not serious	Not serious	Very serious (−2)[b]	Undetected	RR 0.70 (0.09–5.64)	13 fewer per 1,000 (41 fewer to 208 more)	⊕◯◯◯ Very low
**Ulcer rebleeding within 7 days**	6 RCTs (n = 1,138)	Serious (−1)[c]	Not serious	Not serious	Very serious (−2)[c]	Undetected	RR 1.07 (0.66–1.72)	6 more per 1,000 (30 fewer to 65 more)	⊕◯◯◯ Very low
**Need for surgical intervention**	11 RCTs (n = 1,632)	Serious (−1)[d]	Not serious	Not serious	Serious (−1)[d]	Undetected	RR 1.42 (0.80–2.53)	8 more per 1,000 (4 fewer to 30 more)	⊕⊕◯◯ Low
**All-cause mortality**	11 RCTs (n = 1,634)	Serious (−1)[e]	Not serious	Not serious	Serious (−1)[e]	Undetected	RR 1.76 (0.90–3.44)	10 more per 1,000 (1 fewer to 33 more)	⊕⊕◯◯ Low
**Mean number of blood units transfused**	7 RCTs (n = 1,288)	Serious (−1)[f]	Not serious	Not serious	Not serious	Undetected	—	MD 0.37 units higher (0.06 lower to 0.80 higher)	⊕⊕⊕◯ Moderate
**Length of hospital stay**	7 RCTs (n = 1,063)	Serious (−1)[g]	Not serious	Not serious	Not serious	Undetected	—	MD 0.35 days longer (0.12 shorter to 0.82 longer)	⊕⊕⊕◯ Moderate

Note. All bodies of evidence began at high certainty because only randomized controlled trials were included. For dichotomous outcomes, absolute effects were calculated using the risk in the intermittent-regimen group as the baseline risk. A threshold of RR < 0.75 or RR > 1.25 was used to identify clinically important benefit or harm; thresholds of ±1 unit and ±1 day were used for mean blood units transfused and hospital stay, respectively.

[a] Downgraded one level for risk of bias because two of the 11 trials were at high risk, eight raised some concerns, and only one was at low risk. Downgraded two levels for very serious imprecision because only 148 events occurred, the 95% CI included clinically important benefit and harm, and the optimal information size was not met. Downgraded one level for suspected publication bias because funnel-plot asymmetry was observed and Egger's regression test was statistically significant (p = 0.033).

[b] Downgraded one level for risk of bias because all three trials raised some concerns in the outcome-specific RoB 2 assessment. Downgraded two levels for very serious imprecision because only 35 events occurred, the extremely wide 95% CI included clinically important benefit and harm, and the optimal information size was not met.

[c] Downgraded one level for risk of bias because all six contributing trials raised some concerns. Downgraded two levels for very serious imprecision because only 105 events occurred, the 95% CI included clinically important benefit and harm, and the optimal information size was not met.

[d] Downgraded one level for risk of bias because two of the 11 trials were at high risk, eight raised some concerns, and only one was at low risk. Downgraded one level for imprecision because only 40 events occurred, the optimal information size was not met, and the 95% CI included no effect and clinically important harm, although it did not include the prespecified threshold for clinically important benefit.

[e] Downgraded one level for risk of bias because one of the 11 trials was at high risk, nine raised some concerns, and only one was at low risk. Downgraded one level for imprecision because only 32 deaths occurred, the optimal information size was not met, and the 95% CI included no effect and clinically important harm, although it did not include the prespecified threshold for clinically important benefit.

[f] Downgraded one level for risk of bias because all seven contributing trials raised some concerns. No downgrade for imprecision was applied because the 95% CI remained within the prespecified threshold of ±1 blood unit.

[g] Downgraded one level for risk of bias because six of the seven contributing trials raised some concerns and only one was at low risk. No downgrade for imprecision was applied because the 95% CI remained within the prespecified threshold of ±1 day.

Abbreviations: CI, confidence interval; GRADE, Grading of Recommendations Assessment, Development and Evaluation; MD, mean difference; RCT, randomized controlled trial; RoB 2, revised Cochrane risk-of-bias tool for randomized trials; RR, risk ratio.
